# Global mRNA decay and 23S rRNA fragmentation in *Gluconobacter oxydans* 621H

**DOI:** 10.1186/s12864-018-5111-1

**Published:** 2018-10-16

**Authors:** Angela Kranz, Andrea Steinmann, Ursula Degner, Aliye Mengus-Kaya, Susana Matamouros, Michael Bott, Tino Polen

**Affiliations:** 1 IBG-1: Biotechnology, Institute of Bio- and Geosciences, Forschungszentrum Jülich GmbH, 52425 Jülich, Germany; 2 The Bioeconomy Science Center (BioSC), c/o Forschungszentrum Jülich GmbH, 52425 Jülich, Germany

**Keywords:** *Gluconobacter oxydans*, mRNA decay, ATP synthase, Tricarboxylic acid cycle, Ribosome, 23S rRNA fragmentation, Intervening sequence

## Abstract

**Background:**

*Gluconobacter oxydans* is a strictly aerobic Gram-negative acetic acid bacterium used industrially for oxidative biotransformations due to its exceptional type of catabolism. It incompletely oxidizes a wide variety of carbohydrates regio- and stereoselectively in the periplasm using membrane-bound dehydrogenases with accumulation of the products in the medium. As a consequence, only a small fraction of the carbon and energy source enters the cell, resulting in a low biomass yield. Additionally, central carbon metabolism is characterized by the absence of a functional glycolysis and absence of a functional tricarboxylic acid (TCA) cycle. Due to these features, *G. oxydans* is a highly interesting model organism. Here we analyzed global mRNA decay in *G. oxydans* to describe its characteristic features and to identify short-lived mRNAs representing potential bottlenecks in the metabolism for further growth improvement by metabolic engineering.

**Results:**

Using DNA microarrays we estimated the mRNA half-lives in *G. oxydans*. Overall, the mRNA half-lives ranged mainly from 3 min to 25 min with a global mean of 5.7 min. The transcripts encoding GroES and GroEL required for proper protein folding ranked at the top among transcripts exhibiting both long half-lives and high abundance. The F-type H^+^-ATP synthase transcripts involved in energy metabolism ranked among the transcripts with the shortest mRNA half-lives. RNAseq analysis revealed low expression levels for genes of the incomplete TCA cycle and also the mRNA half-lives of several of those were short and below the global mean. The mRNA decay analysis also revealed an apparent instability of full-length 23S rRNA. Further analysis of the ribosome-associated rRNA revealed a 23S rRNA fragmentation pattern exhibiting new cleavage regions in 23S rRNAs which were previously not known.

**Conclusions:**

The very short mRNA half-lives of the H^+^-ATP synthase, which is likely responsible for the ATP-proton motive force interconversion in *G. oxydans* under many or most conditions, is notably in contrast to mRNA decay data from other bacteria. Together with the short mRNA half-lives and low expression of some other central metabolic genes it could limit intended improvements of *G. oxydans’* biomass yield by metabolic engineering. Also, further studies are needed to unravel the multistep process of the 23S rRNA fragmentation in *G. oxydans*.

**Electronic supplementary material:**

The online version of this article (10.1186/s12864-018-5111-1) contains supplementary material, which is available to authorized users.

## Background

*Gluconobacter oxydans* is a Gram-negative, strictly aerobic acetic acid bacterium industrially used for oxidative biotransformations of carbohydrates. Important products are e.g. L-sorbose, a precursor for vitamin C production, dihydroxyacetone, a chemical used for tanning lotions, or 6-amino-L-sorbose, a precursor of the antidiabetic drug miglitol [1–6]. The beneficial ability of *G. oxydans* is the incomplete oxidation of a variety of substrates (e.g. sugars and sugar alcohols) in the periplasm by membrane-bound dehydrogenases and release of resulting products into the cultivation medium [7–9]. Correspondingly, only a small amount of substrate is taken up by the cell and channeled into the cytoplasmic metabolism for growth [10]. Genome sequencing revealed the absence of genes coding for enzymes of the central metabolism, such as 6-phosphofructokinase, succinyl-CoA synthetase, and succinate dehydrogenase [11]. Accordingly, the Embden-Meyerhof-Parnas pathway (glycolysis) and the tricarboxylic acid (TCA) cycle are incomplete. Both the dominant incomplete periplasmic oxidation and the incomplete cytoplasmic sugar metabolism contribute to the limited assimilation of carbohydrates into new cell material and therefore to a low biomass yield. Industrial use of *G. oxydans* for oxidative biotransformations is therefore costly. To overcome these hindrances, metabolic engineering was performed to complete the TCA cycle by introducing heterologous genes for succinate dehydrogenase and succinyl-CoA synthetase into the genome with simultaneous deletion of the genes for the membrane-bound and soluble glucose dehydrogenase, thus abolishing periplasmic and cytoplasmic glucose oxidation [12, 13]. Furthermore, the NADH oxidation capacity was increased by introducing an additional NADH dehydrogenase gene [14]. These steps led to an increase of the biomass yield on glucose by up to 60%, thereby reducing the costs for biomass formation. Although this is already very advantageous for industrial applications, other still unrecognized bottlenecks in *G. oxydans’* naturally evolved partially incomplete metabolism might exist.

Therefore, in this study we conducted genome-wide mRNA decay analysis to get further insights into the physiology of *G. oxydans*. In living cells, the abundance of mRNAs is a result of the balance between gene expression and degradation of mRNAs. Global mRNA decay analysis in prokaryotes was already described, for example, for the model microorganisms *Escherichia coli* [15], *Bacillus subtilis* [16], and *Mycobacterium tuberculosis* [17]. These studies used rifampicin for inhibition of transcription at different time points during growth to measure the changes of relative mRNA levels using DNA microarrays. These changes reflect the degradation of transcripts and allow the calculation of mRNA half-lives [15, 18]. In bacteria, mRNA half-lives typically range from around 1 min or shorter up to 30 min [15, 16, 19]. Generally, a correlation between the half-lives of transcripts and the cellular function of the encoded proteins was observed in these studies. Transcripts associated to housekeeping functions such as cell envelope and ion transport exhibited relatively long mRNA half-lives, whereas genes involved in stress responses and in regulatory functions exhibited a faster transcript turnover to adapt to environmental changes in a short time [20, 21]. Likewise, mRNA half-lives are not fixed and can be affected by, for example, alterations in the growth rate when environmental conditions change [22, 23]. Since mRNA half-lives are affected by different growth conditions, regulation of their stability and degradation is quite diverse. It is dependent on secondary structures of the 5′ and 3′ untranslated regions, posttranscriptional modifications such as polyadenylation, abundance of ribonucleases, presence of cleavage sites recognized by ribonucleases, interaction with small regulatory RNAs, as well as location of the mRNAs in the cell [20, 24].

Here, we measured temporal RNA level changes in *G. oxydans* 621H in response to rifampicin and estimated the mRNA half-lives. The mRNA decay analysis additionally revealed an apparent instability of the full-length 23S rRNA. Enrichment of ribosomes and further analysis of the associated rRNAs uncovered that the 23S rRNA is fragmented in *G. oxydans*.

## Methods

### Bacterial strain and cultivation conditions

In this study, the wild type *Gluconobacter oxydans* 621H strain (DSM 2343) from the German Collection of Microorganisms and Cell Cultures (DSMZ) was used. The cells were cultivated in mannitol medium containing 220 mM (4% *w*/*v*) mannitol, 5 g L^− 1^ yeast extract, 1 g L^− 1^ KH_2_PO_4_, 1 g L^− 1^ (NH_4_)_2_SO_4_, 2.5 g L^− 1^ MgSO_4_ × 7 H_2_O, and 50 μg mL^− 1^ cefoxitin as antibiotic. Cells were grown in 500 mL shaking flasks with three baffles containing 50 mL of the mannitol medium (30 °C, 140 rpm). Cell growth in liquid culture was followed by measuring the optical density at 600 nm (OD_600_) using a spectrophotometer (UV-1800, Shimadzu). If rifampicin was applied, it was added to a cell culture with an OD_600_ of 0.6 to 0.8 from a stock solution (50 mg mL^− 1^ in methanol) to obtain the final concentration as indicated, while the appropriate volume of methanol without rifampicin was added to the control culture. For isolation of total RNA from cells and purification of ribosomes, cells were harvested at the indicated OD_600_ as described below and stored at − 20 °C until use [10].

### Isolation of RNA

For determination of mRNA half-lives by DNA microarray analysis, total RNA was isolated from harvested cells as described [10]. The RNA fraction of ribosomes was isolated by phenol-chloroform-isoamyl alcohol (25:24:1) and chloroform-isoamyl alcohol (24:1) extractions followed by ethanol precipitation [25]. RNA concentrations were determined photometrically using a Nanodrop ND-1000 and fluorometrically using a Qubit® 2.0 device and the Qubit® RNA BR Assay Kit (Life Technologies). RNA samples were quality-checked and visualized on formaldehyde agarose gels as described [26].

### DNA microarray analysis

The DNA microarray analysis aimed at the determination of the time-dependent mRNA level changes in *G. oxydans* after addition of rifampicin. The analysis was performed three times with independent cultures for each time point. *G. oxydans* cells were harvested for isolation of total RNA directly before (t0) and 2 min (t2), 5 min (t5), 10 min (t10) as well as 15 min (t15) after addition of rifampicin. Each RNA sample isolated after addition of rifampicin (tx) was compared to the t0 RNA sample. For pairwise comparisons, the A mix and the B mix of the Agilent Spike-In Kit (Agilent Technologies) was used to spike t0 and tx RNA samples accordingly. The synthesis of labeled cDNA from the RNA samples was carried out as described [10]. Custom-made 4 × 44 K DNA microarrays for genome-wide gene expression analysis were obtained from Agilent Technologies and were designed using Agilent’s eArray platform (https://earray.chem.agilent.com/earray). The array design comprised oligonucleotides for the annotated protein-coding genes and the structural RNA genes of *G. oxydans* 621H (CP000009, and CP000004 to CP000008) [11], as well as Agilent’s control spots. After hybridization according to the manufacturer’s instructions, the arrays were washed using Agilent’s wash buffer kit. Subsequently, the fluorescence of DNA microarrays was determined at 532 nm (Cy3-dUTP) and 635 nm (Cy5-dUTP) at 5 μm resolution with a GenePix 4000B laser scanner and GenePix Pro 6.0 software (Molecular Devices). Raw data files of fluorescence images were saved in TIFF format followed by quantitative image analysis (GenePix Pro 6.0) using the corresponding Agilent’s gene array list (GAL) file. The results were saved as GPR file containing the non-normalized ratio of median values (GenePix Pro 6.0).

### Data normalization and calculation of mRNA half-lives

For calculation of mRNA half-lives, first the ratio of median values (GenePix Pro 6.0) reflecting the relative mRNA level changes were normalized using a factor. This factor was calculated for each hybridization based on the log base 2 of the non-normalized ratio of median values of the Spike-In (+)E1A_r60_1 and (+)E1A_r60_a20 RNAs each having 32 array spots randomly scattered (Agilent Technologies). Both RNAs are present in mix A and mix B in a 1:1 ratio A/B (Agilent Technologies). Accordingly, the normalization factor was calculated that the log base 2 of the normalized ratio of median values of the 1:1 control RNAs is 0 on average. Subsequently, the ratio of median value for each gene was normalized with the calculated factor. All microarray data including the normalized ratio of medians were stored for further analysis, quality filtering and mRNA half-life calculation in the in-house DNA microarray database [27]. A normalized ratio value was included in the calculation of the average of the triplicates for each time point if the following quality filter was fulfilled by the spot data (GenePix Pro 6.0): i) Flags ≥0 and ii) signal/noise ≥3 for Cy5 (F635Median / B635Median) or Cy3 (F532Median / B532Median). The resulting data matrix with the average values of the four time points was used to calculate the mRNA half-lives and R^2^ in Excel (Microsoft) by linear fit as described for *E. coli* data [18]. For further analysis, genes were functionally grouped according to the assigned product functions [11]. Significant differences between functional groups were identified *via* a one-way ANOVA test using Excel (Microsoft). Subsequently, a *post-hoc* t-test was performed to identify mean mRNA half-lives of functional groups, which differ significantly from the overall average half-life. *p* values were adjusted using Bonferroni correction, which allows adjustment of *p* values after multiple comparisons [28].

### Determination of FPKM expression values

For determination of FPKM values reflecting mRNA abundance, we used FASTQ files that were generated previously for comprehensive RNAseq analysis of *G. oxydans* (NCBI accession number: ERR2232412) [29]. Sequencing reads were trimmed and strand-specifically mapped to the genome reference (CP000009) and the five plasmids pGOX1 to pGOX5 (CP000004 – CP000008) using the RNAseq analysis tool of the CLC Genomics Workbench (Qiagen Aarhus A/S) to determine absolute FPKM values [30]. Only uniquely mapped reads with ≤1% mismatches were considered for this analysis. Linear regression analysis of transcript abundances and mRNA half-lives was performed with GraphPad Prism 7.00 using default settings.

### Purification of ribosomes

For preparation of lysates, frozen cell pellets were thawed and resuspended to 0.2 g mL^− 1^ in lysis buffer (70 mM KCl, 10 mM MgCl_2_, 10 mM Tris-HCl, pH 7.4). Resuspended cells were disrupted in a French press (SLM Aminco) at 15,000 psi (3 passages) using 10 mL of cell suspension. Remaining intact cells and debris were removed by centrifugation (20,000 *g*; 20 min; 4 °C) and the supernatant was filtered through a 0.22 μm filter. The protein concentration was determined using Pierce BCA protein assay kit (Thermo Fisher Scientific). If required, the volume was adjusted by addition of lysis buffer to obtain a concentration of 1.5 to 4.5 mg_protein_ mL^− 1^. Ribosomes were purified based on the use of a strong anion exchange quarternary amine (QA) monolithic column [31]. Therefore, 0.5 mL of the prepared cell lysate was injected into an ÄKTA pure FPLC system (GE Healthcare Life Sciences) equipped with two CIM® QA-0.34 mL monolithic disks (BIA separations) encased within a polyetheretherketone (PEEK) housing and pre-equilibrated in lysis buffer. The chromatography was performed at a flow rate of 2 mL min^− 1^ using lysis buffer (buffer A) and lysis buffer containing 1 M NaCl (buffer B). The loaded QA monolithic disks were washed with 5 column volumes of buffer A and sequentially eluted with 7 column volumes of 40%, 56% and 100% of buffer B. During the step-wise elution the online chromatogram was visually inspected for upcoming peaks to manually collect from the start to the end of a peak into one elution fraction. The collected fractions were further analyzed using mass spectrometry to identify proteins.

### Protein identification

For the identification of proteins in relevant elution fractions, proteins were precipitated with trichloroacetic acid (10%) and resuspended in 50 μL of digestion buffer followed by tryptic digestion using the Trypsin Singles Proteomics Grade Kit according to the manufacturer’s instructions for solution digestion in a volume of 100 μL (Sigma-Aldrich). After tryptic digestion, the peptides were separated chromatographically on a nanoLC Eksigent ekspert™ 425 system (Sciex) coupled with a quartz emitter Tip (New Objective) to a TripleTof™ 6600 mass spectrometer (Sciex). Digested samples were loaded on a pre-column (ChromXP C18–3 μm, 350 μm × 0.5 mm, Sciex) for desalting and enrichment using a flow of 3 μL/min (10 min) of buffer A (0.1% formic acid in HLPC grade water). The separation of peptides followed on an analytical column (ChromXP 3C18-CL-120, 0.075 × 150 mm, Sciex) with a gradient method (125 min) using buffer A and buffer B (0.1% formic acid in acetonitrile) at 40 °C and a flow of 300 nL/min. The gradient conditions were 5% of buffer B for 1 min, 5–9% for 9 min, 9–20% for 50 min, 20–40% for 40 min, 40–80% for 5 min and 80% for 4 min. The mass spectrometer was operated with a “top 50” method: Initially, a 250 ms survey scan (TOF-MS mass range 400–1500 amu, high resolution mode) was collected from which the top 50 precursor ions were automatically selected for fragmentation, whereby each MS/MS event (mass range 100–1700 amu, high sensitivity mode) consisted of a 75 ms fragment ion scan. The source and gas settings were 2200 V spray, 40 psi curtain gas, 6 psi ion source gas, and 75 °C interface heater. The mass spectrometry data obtained were processed with ProteinPilot™ using the Paragon algorithm (V4.5 beta, Sciex) for identification of peptides (99% confidence) and proteins (1% false discovery rate).

### rRNA sequencing

RNA purified from ribosomes was sequenced to analyze the rRNAs. Sequencing libraries were generated using the TruSeq stranded mRNA sample preparation kit (Illumina) according to the manufacturer’s instructions, yet without the fragmentation step. cDNA libraries were quantified and sequenced as described [29]. Sequencing reads were processed and mapped to both the four 23S rRNA genes (GOX0221, GOX1159, GOX1319, and GOX1467) and 16S rRNA genes (GOX0224, GOX1156, GOX1316, and GOX1464) using CLC Genomics Workbench (Qiagen Aarhus A/S). The default mapping parameters were changed to consider only reads, which mapped over their complete length with an identity of at least 99%. The coverage *per* base was extracted and manually inspected in Excel (Microsoft) to identify regions with a coverage < 5% of the average gene coverage and visualized with Origin (OriginLab).

## Results

### Global mRNA half-lives in *G. oxydans* ranged from 2 to 25 min

**Fig. 1:**
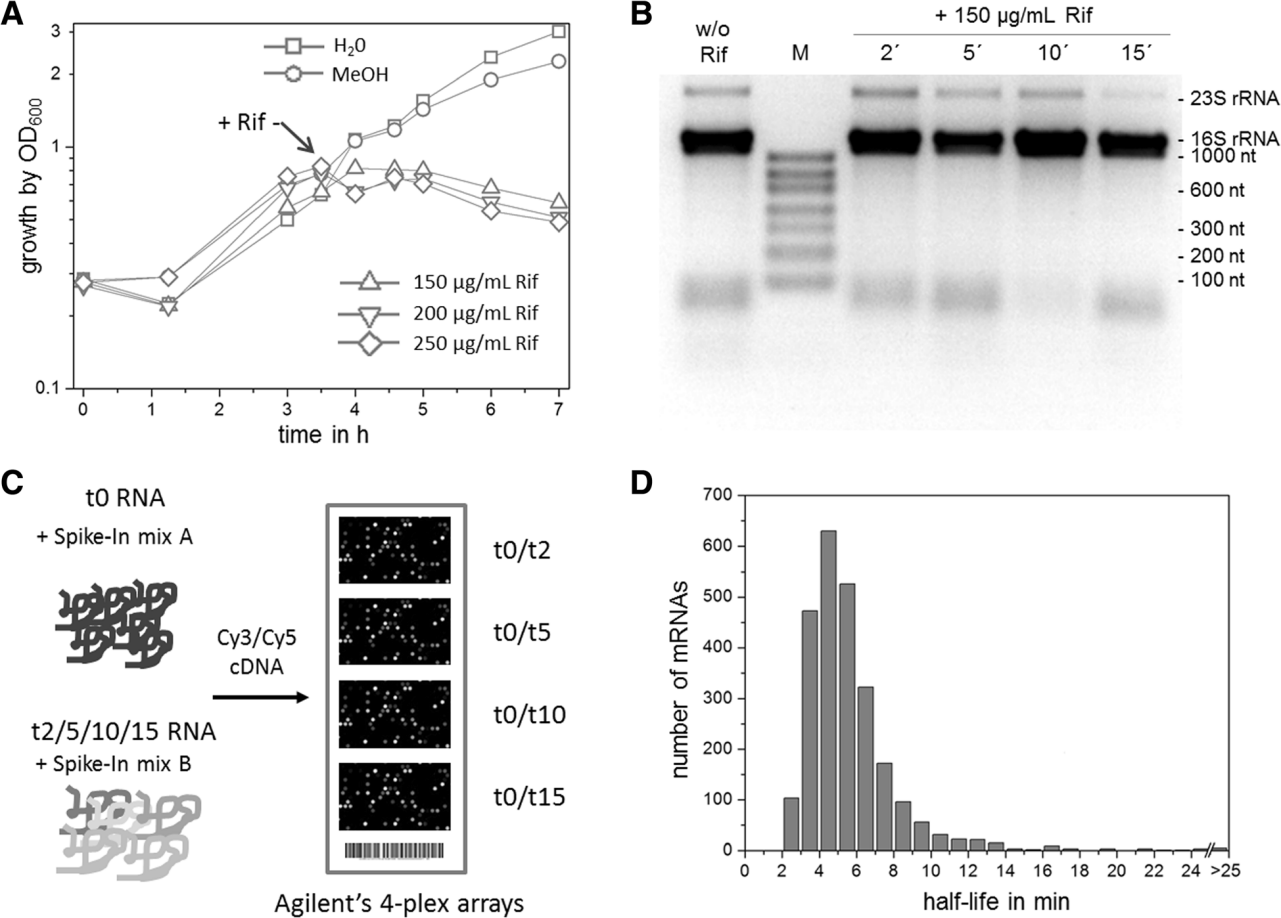
Overview of global mRNA decay analysis. **a** Growth of *G. oxydans* 621H in the presence of different concentrations of rifampicin as well as the controls methanol and water. The addition of rifampicin or a control is indicated by the arrow. **b** Formaldehyde agarose gel (1.8%) analysis to inspect the quality of total RNA isolated from *G. oxydans* cells before and 2, 5, 10, and 15 min after addition of 150 μg/mL of rifampicin. Aliquots of 1 μg of total RNA were loaded. Bands corresponding to the 23S rRNAs (2709 to 2711 nt) and the 16S rRNAs (1478 nt) are indicated as well as the sizes of the RNA fragments present in the RiboRuler Low Range RNA Ladder (M). w/o Rif, without rifampicin. **c** Schema of the experimental design. RNA isolated at the given time points were mixed with Spike-In RNA mix A or B and used for cDNA synthesis and Cy3 or Cy5 labeling. cDNA synthesized from RNA isolated from cells before addition of rifampicin (t0) and 2, 5, 10, and 15 min after addition of 150 μg/mL rifampicin were pairwise mixed and hybridized on Agilent 4-plex arrays. mRNA half-lives were calculated based on three biological replicates including dye-swap labeling. **d** Histogram showing the distribution of calculated mRNA half-lives of 2500 genes from *G. oxydans* where R^2^ was > 0.7 when using all four or the first three sampling times

As outlined in the introduction, *G. oxydans* has an unusual type of metabolism characterized by an incomplete periplasmic oxidation of carbon sources, no glycolysis, lack of a full TCA cycle, and low biomass yield. It thus represents an interesting model organism for the acetic acid bacteria. In a recent study, we employed RNAseq approaches to determine global gene expression, operon structures, transcription start sites, promoter motifs, and ribosome binding sites [29]. Here, we extended these genome-wide studies by determining the mRNA half-lives using DNA microarrays to improve the understanding of gene expression in *G. oxydans*. Typically, mRNA half-lives are determined in rifampicin experiments. Rifampicin inhibits the activity of the RNA polymerase [32], thereby enabling the measurement of the time-dependent decrease of mRNA levels by turnover in the absence of mRNA de novo synthesis. We first tested the influence of different concentrations of rifampicin on the growth of *G. oxydans* in liquid culture. Based on data reported for *Escherichia coli*, *Bacillus subtilis*, *Mycobacterium tuberculosis* and two *Sulfolobus spp.* we focused on a range up to 250 μg/mL [15–17, 19]. With 250 μg/mL and 200 μg/mL of rifampicin a notable drop of the cell density within 30 min was observed, suggesting cell damage and lysis to some extent (Fig. 1a). With 150 μg/mL of rifampicin a growth inhibition within 30 min was observed without a drop of the cell density (Fig. 1a). Although apparently similar results in growth stop were obtained with 100 μg/mL and 50 μg/mL of rifampicin (data not shown) as with 150 μg/mL, we chose 150 μg/mL of rifampicin to analyze the global mRNA decay in *G. oxydans*.

For comparison of transcriptomes, total RNA was isolated from cells directly before addition of rifampicin and 2, 5, 10, and 15 min afterwards. Formaldehyde agarose gels were used to size-separate and visualize the isolated RNA to assess its quality (Fig. 1b). The 23S rRNA band was generally much weaker compared to the 16S rRNA and almost disappeared after 15 min, suggesting a decay or further processing of the expected mature full-length 23S rRNA transcripts with 2709, 2710 and 2711 nt in *G. oxydans* 621H. These results suggest that for *G. oxydans* the rRNAs should better not be used for microarray data normalization, as it was done in other mRNA decay studies where the rRNAs of the host were considered as stable enough to be used for data normalization [18]. We therefore used the Spike-In RNA mixtures A and B (Agilent Technologies), which contain several RNA transcripts for which known ratios between mix A and mix B can be expected. For the pairwise DNA microarray comparisons of transcriptomes corresponding to the time point before addition of rifampicin (t0) and afterwards (t2, t5, t10, and t15), either mix A or B were added accordingly to the RNA samples before cDNA synthesis (Fig. 1c). Spike-In RNA transcripts present in a 1:1 ratio between mix A and B were used to calculate for each microarray hybridization the normalization factor by which all ratios obtained in the respective hybridization experiments were normalized. The normalized ratio data were then used to calculate the average mRNA ratio for each gene at each time point (t0/t2, t0/t5, t0/t10, t0/t15) from three independent biological replicate experiments. Subsequently, the mRNA half-lives can be estimated *via* linear regression of the log ratios over time. Based on the four time points of the analysis and filtering for R^2^ > 0.7 of the linear fit [18], we obtained half-life values for 1193 transcripts. That number corresponds to 44% of all protein-coding genes of *G. oxydans* 621H [11] and is in the range reported for other mRNA decay studies, e.g. for *B. subtilis* (35%), *E. coli* (53%), *M. tuberculosis* (53%), or *S. acidocaldarius* (70%) [15–17, 19]. Nevertheless, there is no half-life estimation for the remaining genes which still represent a significant proportion (56% for *G. oxydans*). The uniform calculation by always including the latest time point may not be adequate for all genes. For example, genes with shorter half-lives may exhibit the decrease in the mRNA level at earlier sampling times, while at later sampling times there is no further decrease for several reasons including inherent limits of the DNA microarray technology used. Therefore, many genes exhibiting this type of mRNA decay kinetics will likely not fulfill a well-intentioned R^2^ criterion for filtering results of the linear regression analysis when later time points are included. Depending on the range of the absolute sampling times it is legitimate to check the outcome of half-life estimations by linear regression with a focus on earlier time points. Accordingly, for the 1446 remaining genes (56%), where R^2^ > 0.7 was not fulfilled when using all sampling times, we omitted the last time point (t0/t15) from half-life calculation. Based on the first three sampling times (t0/t2, t0/t5, t0/t10) further half-life estimations with an R^2^ > 0.7 were obtained for 1307 transcripts. Together, this resulted in mRNA half-life estimations for 2500 (95%) of the protein-coding genes of *G. oxydans* (Additional file 1: Table S1). Overall, the calculated mRNA half-lives of the 2500 genes mainly ranged from approximately 2 min to 25 min. Solely two operons consisting of 8 genes encoding hypothetical and phage-related proteins exhibited apparent mRNA half-lives from 25 to 75 min. The global mean was 5.7 min and the median 5.1 min, which is also reflected by the high number of transcripts exhibiting half-lives between 3 and 7 min (Fig. 1d).

### mRNA half-life characteristics in *G. oxydans*

**Fig. 2:**
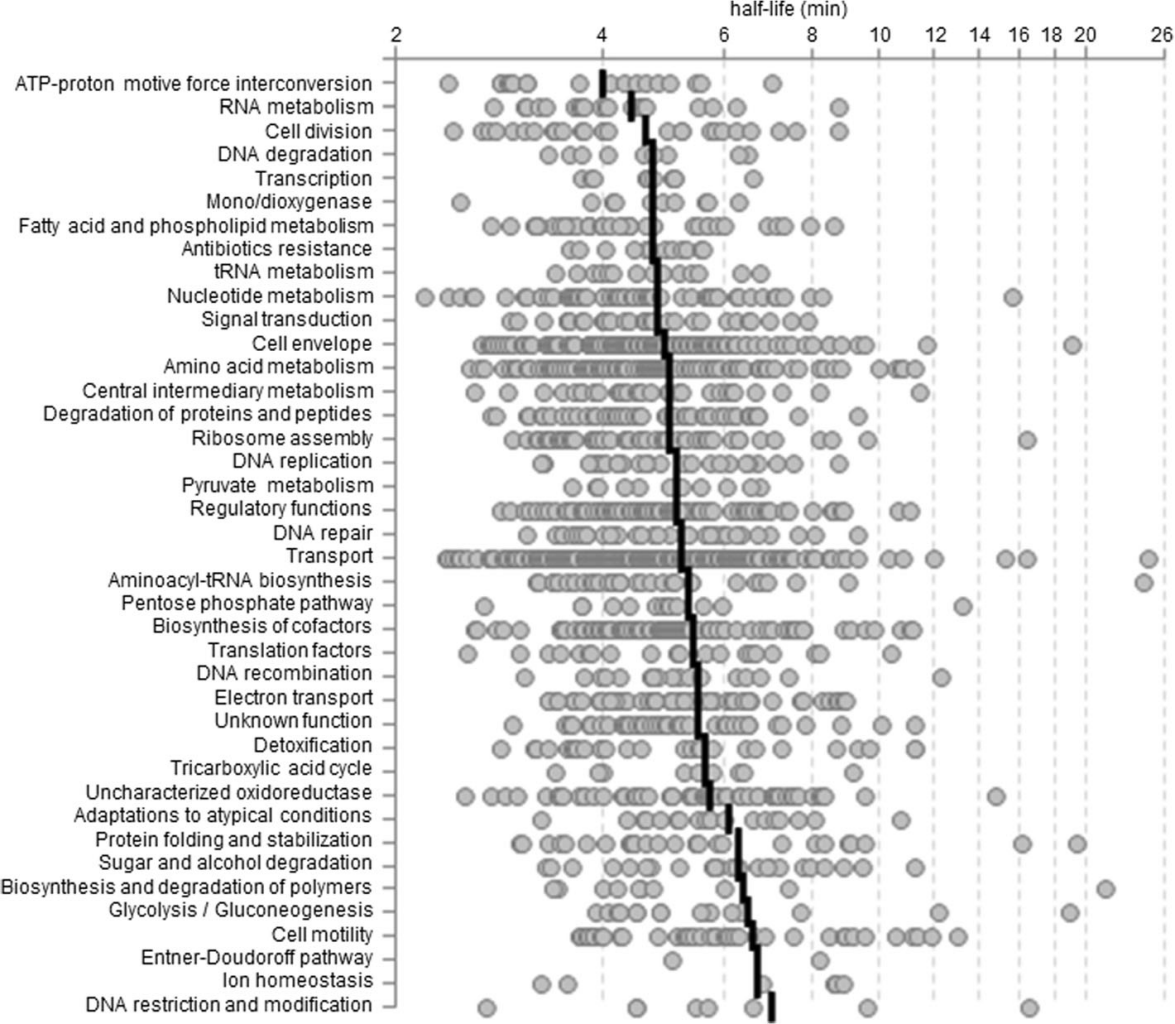
Distribution of mRNA half-lives () with mean value indicator () based on the functional categories or subcategories and number of genes given in Table 1

In previous studies, correlations between the stability of mRNAs and the functional group of the gene products were observed [15, 16, 19]. For *G. oxydans*, one-sided ANOVA test revealed significant differences between functional groups (*p* < 0.0001). Taken into account the within-group variance subsequent *post hoc* analysis identified 2 categories with significantly shorter mean half-lives than the overall average half-life. These categories were ATP-proton motive force interconversion and RNA metabolism (Table 1, Fig. 2).

**Table 1:** Functional categories or subcategories comprising 1582 assigned protein-coding ORFs with mean mRNA half-lives based on data from 1526 (96%) transcripts (Additional file 1: Table S1)

functional category / pathway ^a)^	number of genes			
	assigned ^a)^	with a calculated mRNA half-life	proportion (%)	half-life ^b)^ (min)
ATP-proton motive force interconversion	18	18	100	4.0**
RNA metabolism	23	23	100	4.4*
Cell division	26	26	100	4.6
DNA degradation	9	9	100	4.7
Transcription	13	10	77	4.7
Mono/dioxygenase	10	10	100	4.7
Fatty acid and phospholipid metabolism	36	36	100	4.7
Antibiotics resistance	12	12	100	4.7
tRNA metabolism	13	13	100	4.7
Nucleotide metabolism	59	58	98	4.8
Signal transduction	30	29	97	4.8
Cell envelope	134	131	98	4.9
Amino acid metabolism	120	117	98	5.0
Central intermediary metabolism	33	31	94	5.0
Degradation of proteins and peptides	51	48	94	5.0
Ribosome assembly	67	52	78	5.0
DNA replication	26	26	100	5.1
Pyruvate metabolism	12	11	92	5.1
Regulatory functions	99	94	95	5.1
DNA repair	34	34	100	5.2
Transport	232	227	98	5.2
Aminoacyl-tRNA biosynthesis	33	33	100	5.3
Pentose phosphate pathway	13	13	100	5.3
Biosynthesis of cofactors	85	84	99	5.4
Translation factors	20	20	100	5.4
DNA recombination	18	17	94	5.5
Electron transport	53	53	100	5.5
Unknown function	41	40	98	5.5
Detoxification	30	27	90	5.6
Tricarboxylic acid cycle	9	9	100	5.6
Uncharacterized oxidoreductase	66	63	96	5.7
Adaptations to atypical conditions	19	19	100	6.1
Protein folding and stabilization	28	28	100	6.3
Sugar and alcohol degradation	25	24	96	6.3
Biosynthesis and degradation of polymers	11	10	91	6.4
Glycolysis / Gluconeogenesis	17	16	94	6.5
Cell motility	41	39	95	6.6
Entner-Doudoroff pathway	2	2	100	6.7
Ion homeostasis	6	6	100	6.7
DNA restriction and modification	8	8	100	7.0

^a)^Functional categories and number of genes are based on assigned gene product functions [11]

^b)^Functional categories with significantly shorter or longer mean half-lives than the overall mean half-life (5.7 min) are highlighted by ** (*p* < 0.001) or * (*p* < 0.05)

**Fig. 3:**
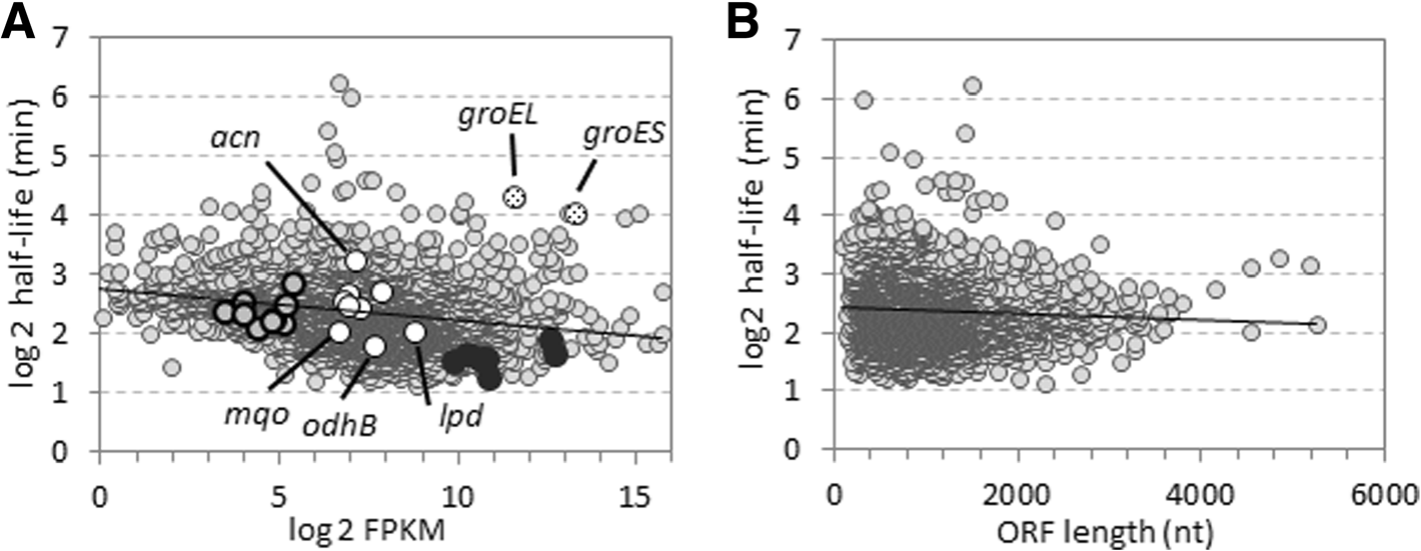
mRNA half-lives versus FPKM expression values and ORF lengths. **a** Linear regression analysis showed a statistically significant slightly negative correlation (*R* = −0.24) between the abundance of transcripts and their half-lives. Plots are related to the half-life data obtained for 2500 transcripts () with R^2^ > 0.7 based on the 4 or 3 sampling times as described. Transcripts of the molecular chaperones GroES (GOX1901) and GroEL (GOX1901) exhibited high expression values as well as long half-lives (). The operons of the F_1_F_o_-type ATP synthase encoded by *atpBEFF*’ (GOX1110–13) and *atpHAGDC* (GOX1310–14) () belong to the operons/genes with the shortest mRNA half-lives in *G. oxydans*. In comparison, the transcripts of the second F_1_F_o_-type ATP synthase encoded by GOX2167–75 () exhibited almost 2-fold longer half-lives and approximately 80-fold lower expression values. Among the genes of the incomplete TCA cycle () the transcript of aconitase of (*acn*, GOX1335) was the most stable, while the transcripts of malate:quinone oxidoreductase (*mqo*, GOX2070), dihydrolipoamide succinyl transferase (E2) of 2-oxoglutarate dehydrogenase (*odhB*, GOX1073), and dihydrolipoamide dehydrogenase (*lpd*, GOX2292) exhibited the shortest mRNA half-lives. FPKM values were obtained with cells grown on mannitol. **b** The mRNA half-lives and ORF lengths of 2500 transcripts () did not correlate (*R* = − 0.06)

According to observations in *E. coli* and the archaeon *Sulfolobus solfataricus*, mRNAs exhibiting higher transcript abundance were on average less stable [15, 19]. In *G. oxydans* we also observed such an inverse relationship (Fig. 3a and Additional file 1: Table S2). On average genes with higher expression values showed slightly shorter apparent mRNA half-lives and *vice versa*. However, certain transcripts exhibited both high abundance and long half-life, such as the transcripts for chaperonin GroEL (GOX1902) and co-chaperonin GroES (GOX1901), nucleoside diphosphate kinase (GOX1927), 50S ribosomal protein L17 (GOX0355), coenzyme PQQ synthesis protein PqqA (GOX0987), as well as a number of putative/hypothetical proteins. On the other hand, certain transcripts exhibited both low abundance and short half-life, such as the transcripts for the cell division proteins FtsE (GOX0273) and FtsX (GOX0274), putative fatty acyl CoA synthetase (GOX1394), ubiquinol-cytochrome c reductase cytochrome b subunit (GOX0566), NAD(P)H-dependent glycerol-3-phosphate dehydrogenase (GOX1880), and cytochrome c subunit and small subunit of membrane-bound aldehyde dehydrogenase (GOX0585, GOX0586). Plotting the mRNA half-lives versus the ORF lengths showed no correlation for *G. oxydans* (Fig. 3b). Similarly, for *E. coli* such a correlation was also not found [15, 33].

Independent of the RNA abundance reflected by the FPKM values, on the individual transcript level the shortest mRNA half-lives were found for GOX1807 (2.2 min) annotated as GTP pyrophosphokinase involved in (p)ppGpp metabolism, for GOX0730, GOX1845, and GOX1426 (2.3 min) encoding hypothetical proteins, for GOX0666 and GOX2394 (2.4 min) involved in ferric iron uptake and purine synthesis, and for GOX1113 (2.4 min) encoding subunit A of an F_1_F_o_-type ATP synthase (Additional file 1: Table S1). This ATP synthase complex is encoded by genes organized in two different operons. We therefore also calculated the average half-lives of all operons (Additional file 1: Table S3) which we recently found based on primary and whole transcriptome data [29]. Among the top 20 operons exhibiting the shortest RNA half-lives were genes encoding proteins involved in iron and manganese homeostasis, one-carbon metabolism, cell shape and cell division proteins, some hypothetical proteins, and notably the F_1_F_o_ ATP synthase encoded by the two operons *atpBEFF*’ (GOX1110–13) and *atpHAGDC* (GOX1310–14) (Table 2). As reported previously, this ATP synthase is an ortholog of the H^+^-translocating ATP synthases of *Acetobacter pasteurianus*, *Gluconacetobacter diazotrophicus* and other α-proteobacteria [34]. *G. oxydans* possesses a second F_1_F_o_-type ATP synthase encoded in one operon (GOX2167–75) which is an ortholog of the Na^+^-translocating F_1_F_o_-type ATP synthases present always in addition to the H^+^-ATP synthase in the archaea *Methanosarcina barkeri* and *M. acetivorans*, in a number of marine and halotolerant bacteria and in pathogenic *Burkholderia* species [35–37]. These genes exhibited on average almost 2-fold longer mRNA half-lives (5.1 min) which is at the global median, yet the apparent expression levels were extremely low (80-fold lower) compared to the H^+^-ATP synthase, which presented transcript levels ranked above the global expression median (Fig. 3a). Thus, the H^+^-ATP synthase with the very short-lived mRNAs (*atpBEFF*’ and *atpHAGDC*) is likely the one which is involved in the energy metabolism under the conditions tested.

**Table 2:** Top 20 operons exhibiting the shortest mRNA half-lives in *G. oxydans* and their assigned gene expression values FPKM. The arrow lines indicate operon genes and genomic orientation.

	locus tag / operon	annotation	operon half-life Ø (min)	half-life (min)	FPKM
|	GOX1310^a^	F_1_F_o_ ATP synthase subunit delta	3.1	3.7	6320
|	GOX1311^a^	F_1_F_o_ ATP synthase subunit alpha		3.0	1780
|	GOX1312^a^	F_1_F_o_ ATP synthase subunit gamma		3.1	1232
|	GOX1313^a^	F_1_F_o_ ATP synthase subunit beta		2.9	1651
↓	GOX1314^a^	F_1_F_o_ ATP synthase epsilon chain		3.1	6950
|	GOX1737	rod shape-determining protein MreB	2.8	2.4	908
|	GOX1738	rod shape-determining protein MreC		2.8	383
↓	GOX1739	hypothetical protein		3.0	93
↑	GOX1110^a^	F_1_F_o_ ATP synthase subunit b’	2.8	2.9	937
|	GOX1111^a^	F_1_F_o_ ATP synthase subunit b		2.9	1600
|	GOX1112^a^	F_1_F_o_ ATP synthase C chain		2.9	1743
|	GOX1113^a^	F_1_F_o_ ATP synthase subunit a		2.4	1911
|	GOX0149	cell division protein MraZ	2.8	3.0	2124
|	GOX0150	SAM-dependent methytransferase		2.8	528
↓	GOX0151	hypothetical protein		2.6	255
↓	GOX1427	FAD-dependent thymidylate synthase	2.7	2.7	1121
↑	GOX0476	putative oxidoreductase	2.7	2.9	658
|	GOX0477	hypothetical protein		2.5	847
|	GOX0478	putative oxidoreductase		2.8	1202
↑	GOX1596	hypothetical protein	2.7	2.7	119
↓	GOX2462	transcriptional regulator	2.7	2.7	4866
↑	GOX0607	D-alanyl-D-alanine carboxypeptidase	2.7	2.7	1540
|	GOX1430	serine protease	2.7	2.8	170
↓	GOX1431	hypothetical protein		2.6	336
|	GOX0024	undecaprenyl pyrophosphate phosphatase	2.6	2.8	198
↓	GOX0025	amino acid permease		2.5	142
↑	GOX2207	methylenetetrahydrofolate reductase	2.6	2.6	547
↓	GOX0649	sugar-proton symporter	2.6	2.6	364
↓	GOX2378	short chain alcohol dehydrogenase	2.6	2.6	301
↑	GOX1151	hypothetical protein	2.5	2.5	2854
↑	GOX1696	hypothetical protein	2.5	2.5	35
↑	GOX2067	manganese transport protein MntH	2.5	2.5	526
↓	GOX0942	hypothetical protein	2.5	2.5	178
↑	GOX1652	heme exporter protein C	2.4	2.4	323
↑	GOX0666	outer membrane receptor for ferric iron uptake	2.4	2.4	222
↑	GOX1426	hypothetical protein	2.4	2.4	577

^a^) For the F_1_F_o_ ATP synthase encoded by two operons the data of the operon GOX1310–14, which was close to the top 20, were also included

A characteristic of *G. oxydans* are the membrane-bound dehydrogenases (mDHs), often with a broad substrate spectrum, incompletely oxidizing a wide variety of carbohydrates in the periplasm with accumulation of the products in the medium [8, 11]. Many of their transcripts exhibited half-lives below the global mean of 5.7 min (Table 3). For the membrane-bound gluconate-2 dehydrogenase mGlDH (GOX1230–2) the longest mDH transcript half-lives were found (8 to 9 min), while expression levels were moderate. Polyol dehydrogenase SldAB (GOX0854–5) required for growth on mannitol and sorbitol, and membrane-bound alcohol dehydrogenase mADH (GOX1067–8) exhibited by far the highest transcript levels. Their transcript half-lives close to 4 min follow the trend of the inverse relationship between FPKM and half-live. The transcripts of the PQQ-dependent mDHs 1 (GOX1857), 3 (GOX1441), and 4 (GOX0516), sorbitol dehydrogenase (GOX2094–97), and aldehyde dehydrogenase mAcDH (GOX0585–7) exhibited very low expression values and half-lives from 4 min to 9 min.

**Table 3:** mRNA half-lives and expression values (FPKM) of genes encoding annotated membrane-bound dehydrogenases. The arrow lines indicate operon genes and genomic orientation.

	locus tag / operon	annotation	half-life (min)	FPKM
↓	GOX0265	membrane-bound glucose dehydrogenase (PQQ)	4.4	146
↑	GOX0516	PQQ-dependent dehydrogenase 4	8.3	22
|	GOX0585	cytochrome c subunit of aldehyde dehydrogenase	3.7	32
|	GOX0586	membrane-bound aldehyde dehydrogenase, small subunit	3.7	38
↓	GOX0587	membrane-bound aldehyde dehydrogenase, large subunit	9.0	31
↑	GOX0854	polyol dehydrogenase subunit SldA	4.2	2147
|	GOX0855	polyol dehydrogenase subunit SldB	4.6	5992
↑	GOX1067	alcohol dehydrogenase cytochrome c subunit precursor	4.0	1807
|	GOX1068	alcohol dehydrogenase large subunit	3.4	1706
↑	GOX1230	gluconate 2-dehydrogenase, cytochrome c subunit	9.0	208
|	GOX1231	gluconate 2-dehydrogenase alpha chain	8.5	791
|	GOX1232	gluconate 2-dehydrogenase gamma chain	7.8	684
↓	GOX1253	D-lactate dehydrogenase	5.0	159
↑	GOX1441	PQQ-dependent dehydrogenase 3	4.7	31
↑	GOX1857	PQQ-containing dehydrogenase 1	7.1	122
|	GOX1968	hypothetical protein	3.6	496
|	GOX1969	alcohol dehydrogenase large subunit	3.5	540
↓	GOX1970	GTP-binding protein EngA	4.3	408
↑	GOX2094	sorbitol dehydrogenase cytochrome c subunit	5.8	10
|	GOX2095	sorbitol dehydrogenase large subunit	5.3	13
|	GOX2096	sorbitol dehydrogenase large subunit	4.1	51
|	GOX2097	sorbitol dehydrogenase small subunit	3.5	74

**Fig. 4:**
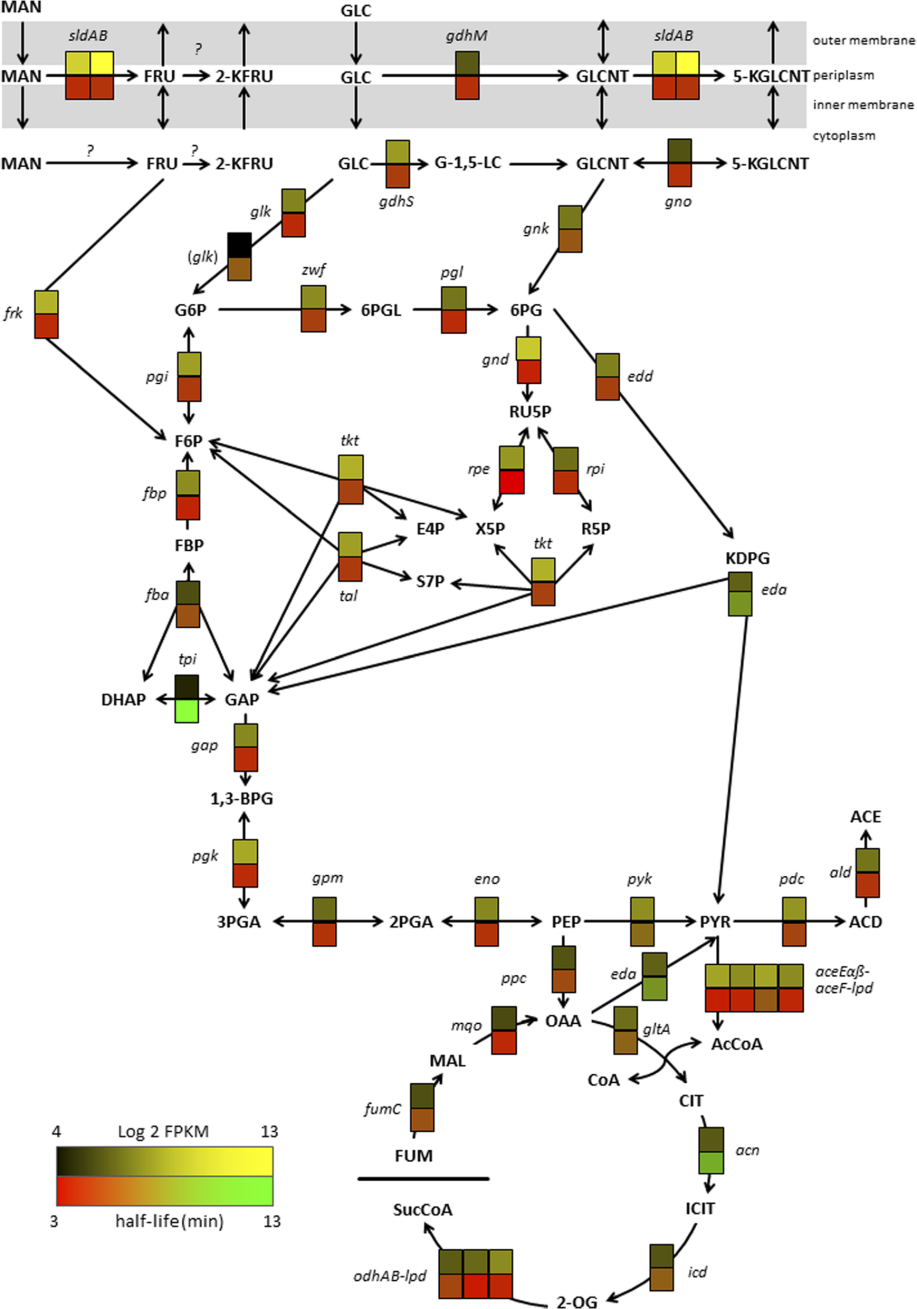
mRNA half-lives and FPKM expression values for genes of the central carbon metabolism. Upper boxes representing a Log2 expression value were colored according to the black-yellow gradient ranging from 4 to 13 (Additional file 1: Table S2). Lower boxes representing a half-life were colored according to the red-green gradient ranging from 3 to 13 min (Additional file 1: Table S1). Genes/Enzymes: *aceEα*, pyruvate dehydrogenase E1 component alpha subunit (GOX2289); *aceEß*, pyruvate dehydrogenase E1 component beta subunit (GOX2290); *aceF*, dihydrolipoamide acetyltransferase component of pyruvate dehydrogenase (GOX2291); *acn*, aconitate hydratase (GOX1335); *ald*, aldehyde dehydrogenase (GOX2018); *eda*, KDPG aldolase (GOX0430); *edd*, phosphogluconate dehydratase (GOX0431); *eno*, enolase (GOX2279); *fba*, fructose-bisphosphate aldolase (GOX0780); *fbp*, fructose 1,6-bisphosphatase (GOX1516); *frk*, fructokinase (GOX0284); *fumC*, fumarate hydratase (GOX1643); *gap*, glyceraldehyde 3-phosphate dehydrogenase (GOX0508); *gdhM*, membrane-bound glucose dehydrogenase (GOX0265); *gdhS*, soluble glucose dehydrogenase (GOX2015); *glk*, glucokinase (GOX2419); (*glk*), putative glucokinase (GOX1182); *gltA*, citrate synthase (GOX1999); *gnd*, 6-phosphogluconate dehydrogenase (GOX1705); *gnk*, gluconokinase (GOX1709); *gno*, gluconate 5-dehydrogenase (GOX2187); *gpm* phosphoglyceromutase (GOX0330); *icd*, isocitrate dehydrogenase (GOX1336); *lpd*, dihydrolipoamide dehydrogenase (GOX2292); *mqo*, malate:quinone oxidoreductase (GOX2070); *odhA*, 2-oxoglutarate dehydrogenase E1 component (GOX0882); *odhB*, dihydrolipoamide succinyltransferase E2 component of 2-oxoglutarate dehydrogenase complex (GOX1073); *pdc*, pyruvate decarboxylase (GOX1081); *pgk*, phosphoglycerate kinase (GOX0507); *pgl*, 6-phosphogluconolactonase (GOX1707); *ppc*, phosphoenolpyruvate carboxylase (GOX0102); *pyk*, pyruvate kinase (GOX2250); *rpe*, ribulosephosphate 3-epimerase (GOX1352); *rpi*, ribose 5-phosphate isomerase (GOX1708); *sldA*, polyol dehydrogenase subunit SldA (GOX0854); *sldB*, polyol dehydrogenase subunit SldB (GOX0855); *tal*/*pgi*, bifunctional transaldolase (GOX1704); *tkt*, transketolase (GOX1703); *tpi*, triosephosphate isomerase (GOX2217); *zwf*, glucose-6-phosphate 1-dehydrogenase (GOX0145). Metabolites: 1,3-BPG, 1,3-bisphosphoglycerate; 2-KFRU, 2-ketofructose; 2-OG, 2-oxoglutarate; 2PGA, 2-phosphogylcerate; 3PGA, 3-phosphoglycerate; 5-KGLCNT, 5-ketogluconate; 6PG, 6-phosphogluconate; 6PGL, 6-phosphogluconolactone; ACD, acetaldehyde; ACE, acetate; AcCoA, acetyl coenzyme A; CIT, citrate; CoA, coenzyme A; DHAP, dihydroxyacetone phosphate; E4P, erythrose 4-phosphate; F6P, fructose 6-phosphate; FBP, fructose 1,6-bisphosphate; FRU, fructose; FUM, fumarate; G-1,5-LC, glucono-1,5-lactone; G6P, glucose 6-phosphate; GAP, glyceraldehyde 3-phosphate; GLC, glucose; GLCNT, gluconate; ICIT, isocitrate; KDPG, 2-keto-3-deoxy-6-phosphogluconate; MAL, malate; MAN, mannitol; OAA, oxaloacetate; PEP, phosphoenolpyruvate; PYR, pyruvate; R5P, ribose 5-phosphate; RU5P, ribulose 5-phosphate; S7P, sedoheptulose 7-phosphate; SucCoA, succinyl coenzyme A; X5P, xylulose 5-phosphate

Since *G. oxydans* has an unusual central metabolism, we were also interested in the stability of transcripts encoding enzymes of the central carbon metabolism including the pentose phosphate pathway (PPP), the Entner-Doudoroff pathway (EDP), and the pyruvate metabolism. Therefore, we mapped the estimated mRNA half-lives to the central carbon metabolism [38]. Overall, mRNA half-lives ranged from 3.6 min for *aceEα* (GOX2289) encoding pyruvate dehydrogenase E1 component subunit α to 12.4 min for one of two annotated triosephosphate isomerases (*tpi*, GOX2217) (Fig. 4). According to the FPKM values this *tpi* transcript showed almost the lowest abundance for genes involved in central carbon metabolism (Fig. 4, Additional file 1: Table S2). This apparently very low expression might be compensated by the longer mRNA half-life. Besides this *tpi* transcript, which is among the top 3% with the longest mRNA half-lives overall, the transcript for dihydroxyacetone kinase (GOX2222), which is linked to central carbon metabolism *via* the product dihydroxyacetone phosphate (DHAP), is with 19.1 min among the top 1% of the most-stable transcripts in *G. oxydans*. The transcripts of the PPP genes exhibited half-lives below the global mean ranging from 3.8 min to 5.1 min. Besides the *rpi* transcript (GOX1708, 4.4 min) encoding ribose-5-phosphate isomerase, the transcript of GOX2218 also annotated to encode a ribose-5-phosphate isomerase, yet without strong sequence similarity to the GOX1708 protein, exhibited a 3-fold longer half-life (13.3 min). The two EDP gene transcripts exhibited a half-life of 5 min (*edd*) and 8.3 min (*eda*). Genes of the incomplete TCA cycle exhibited the lowest apparent expression in the central metabolism based on the FPKM values, with the aconitase transcript (GOX1335) as the most stable (9.3 min) above the global mean, and with the transcripts of *odhB* (GOX1073, 3.4 min) encoding dihydrolipoamide succinyl transferase (E2) of the 2-oxoglutarate dehydrogenase, *mqo* (GOX2070, 4 min) encoding malate:quinone oxidoreductase, and *lpd* (GOX2292, 4 min) encoding dihydrolipoamide dehydrogenase exhibiting the shortest half-lives (Fig. 3a, Fig. 4, Additional file 1: Table S2). Expression of *ppc* and *eda* encoding phosphoenolpyruvate carboxylase and KDPG aldolase, respectively, also appeared to be rather low. PPC is typically involved in anaplerosis, which is important to replenish intermediates that have been extracted for biosynthesis from a functional TCA cycle. *G. oxydans* lacks a full TCA cycle and anaplerotic PPC may be not important in *G. oxydans* wild type. In summary, the genes involved in central carbon metabolism exhibited a relatively broad range of mRNA half-lives. Shorter half-lives and rather low FPKM expression values of some genes may yield a low protein level and therefore a low enzymatic activity possibly representing potential bottlenecks for a high carbon flux in the central metabolism of *G. oxydans*.

### Mapping analysis revealed fragmentation regions in 23S rRNAs from *G. oxydans*

During the global mRNA decay analysis the visualization results of total RNA samples indicated that the full-length 23S rRNA from *G. oxydans* was underrepresented and appeared very unstable compared to the 16S rRNA (Fig. 1a). In the genome of *G. oxydans* 621H four rRNA operons are present [11]. In these operons, GOX0224, GOX1156, GOX1316 and GOX1464 encode for sequence-identical 16S rRNA transcripts with 1478 nt. GOX0221 and GOX1467 encode sequence-identical 23S rRNA transcripts with 2710 nt. The C at position 2119 is absent in the 23S rRNA transcript of GOX1159 with 2709 nt. At position 1746 a C is inserted in the 23S rRNA transcript of GOX1319 with 2711 nt. The underrepresentation of processed full-length 23S rRNA in *G. oxydans* RNA samples always raised questions on the quality of the prepared RNA, for example in DNA microarray studies. To check whether the 23S rRNA in *G. oxydans* maybe indeed rapidly degraded or rather is fragmented as found in several bacteria including α-proteobacteria other than *Gluconobacter* [39, 40], we analyzed the rRNA fraction obtained from *G. oxydans* ribosomes. Therefore, we chromatographically enriched ribosomes from *G. oxydans* cells grown in mannitol medium without rifampicin. We analyzed two time points, one in the exponential growth phase (OD_600_ = 1 after 4.25 h of cultivation) and one in the early stationary phase (OD_600_ = 2.5 after 9.5 h of cultivation). Cell pellets were used to obtain crude protein extracts. The sample volumes were adjusted to obtain the protein concentration suitable for the chromatography runs on the monolithic disks. According to the elution profile we always obtained four major peaks termed P1 to P4 (Additional file 1: Figure S1A). The sum of the protein content in the elution fractions of the four peaks typically comprised 85% to 95% of the total protein applied to the column (Additional file 1: Table S4). According to nanoLC-based mass spectrometry, 933 *G. oxydans* proteins were identified overall in the four protein fractions (Additional file 1: Table S5). 449 to 865 proteins were identified in peaks P1 and P2, with the apparent overall content of ribosomal proteins being rather low. The highest abundance of ribosomal proteins was found in peak P3 as judged by SDS-PAGE analysis and MALDI-ToF mass spectrometry (data not shown) as well as by the overall high numbers of detected peptides *per* ribosomal protein in the nanoLC-MS/MS analysis (Additional file 1: Table S6). Moreover, by far the largest amount of RNA could be isolated from the protein fraction of peak P3 (Additional file 1: Table S4), which is expected when ribosomes are enriched according to the method used. All 30S ribosomal proteins were detected in peak P3 (Additional file 1: Table S6). From the 50S ribosomal proteins, the protein L32 (GOX0117), L36 (GOX0732), and L34P (GOX1825) could not be detected at all. Overall, the chromatogram and elution profile of ribosomes in peak P3 is typical for this method as already described elsewhere for other bacteria [31].

**Fig. 5:**
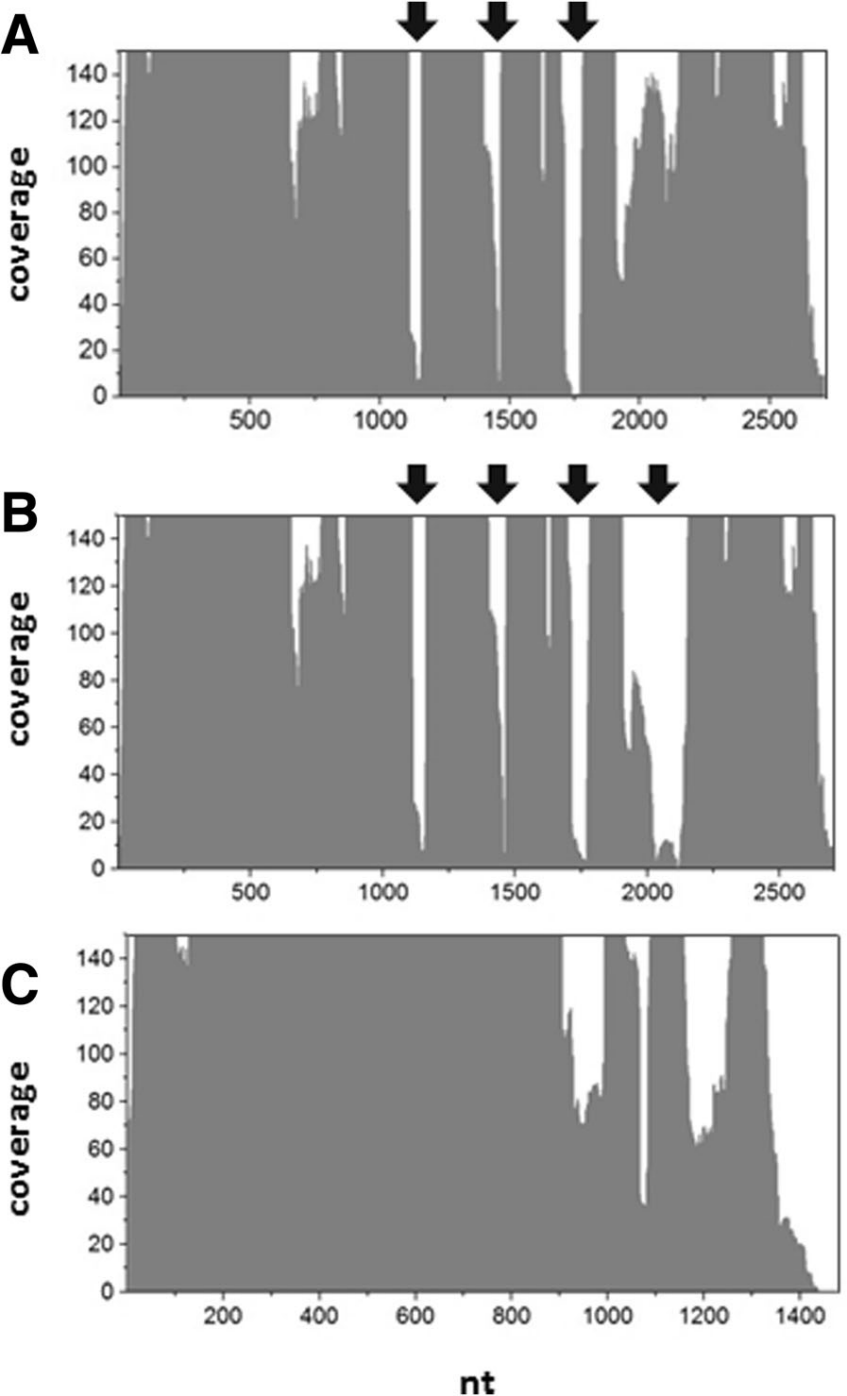
Graphical representation of the 23S rRNA (**a**, **b**) and 16S rRNA (**c**) mapping coverage. The mapping is based on the Illumina reads obtained with RNA samples isolated from enriched ribosomes (peak P3) from cells in the exponential growth phase. The y axes are zoomed to a maximum of 150 to better illustrate the very low coverage regions (< 5%) indicated by the arrows. **a** Mapping coverage of the 23S rRNA locus GOX1319 (2711 nt). The same coverage pattern was obtained for the 23S rRNA loci GOX0221 and GOX1467 (2710 nt). **b** Mapping coverage of the 23S rRNA locus GOX1159 (2709 nt) which indicates an additional fragmentation site. **c** Mapping coverage obtained for the 16S rRNA locus GOX1156. The same coverage pattern was observed for the other 16S rRNA loci GOX0224, GOX1316, and GOX1464

In the RNA isolated from peak P3 a mature full-length 23S rRNA could not be detected in the formaldehyde agarose gel analysis at ~ 2710 nt, yet the RNA samples exhibited a specific pattern of smaller fragments (Additional file 1: Figure S1B). This suggested that the 23S rRNA is fragmented in *G. oxydans* ribosomes. The pattern showed three fragments at approximately 1500 nt, 900 nt and 800 nt, and two shorter fragments at approximately 300 nt and 400 nt. By size the longest fragment of ~ 1500 nt corresponds to the 16S rRNA. To narrow down the regions of fragmentation in the 23S RNA transcripts, the ribosome-associated RNA was sequenced *via* next-generation sequencing and the paired-end reads were mapped to the sequences of the 16S and 23S rRNA gene loci from *G. oxydans* using very stringent mapping parameters. To identify possible fragmentation positions based on the mapping coverage we searched for regions with less than 5% coverage of the average coverage for the entire gene locus (Fig. 5). In the RNA samples from the exponential phase, three regions with such a low or almost no coverage were found for the 23S rRNA transcripts suggesting fragmentation sites in GOX0221, GOX1319, and GOX1467. The same three regions were also found in the shortest 23S rRNA (GOX1159; 2709 nt), but in this case a fourth potential fragmentation region was present according to the very low mapping coverage (Fig. 5b). The three very low coverage regions found for GOX1159 were at positions 1140–1159 (20 nt), 1456–1465 (10 nt), and 1717–1770 (55 nt) (Fig. 5, Additional file 1: Tables S7-S10). For the other three 23S rRNA genes, the positions of two of the three regions differed by maximal two nucleotides: GOX0221 (1139–1160; 1456–1465; 1716–1770), GOX1319 (1140–1159; 1456–1465; 1715–1771), GOX1467 (1140–1159; 1456–1465; 1717–1770). The fourth region only found for GOX1159 was at position 2022–2136 (Additional file 1: Table S8). In this fourth region GOX1159 exhibits two nucleotide differences compared to the other three 23S rRNA genes at nt 2034 and nt 2119. The absence of mapping coverage in this region raises the question whether GOX1159 was actually transcribed into RNA. It should be mentioned that GOX1159 exhibits another nucleotide difference at nt 936 (T). According to the mapped reads 871-fold (Additional file 1: Table S8) and 8301-fold (Additional file 1: Table S16) coverage/reads were obtained at nt 936 in GOX1159. This shows the presence of the T and suggests that GOX1159 was very likely transcribed into RNA. For the 16S rRNA genes we did not find such low coverage regions indicating the presence of the mature full-length transcripts in the ribosomes (Fig. 5c, Additional file 1: Tables S11-S14). In the RNA samples from the stationary phase, the Illumina read mappings showed very similar results (Additional file 1: Tables S15-S22). Only for the second and fourth fragmentation position an extension of the low coverage regions by 55 nt (1401–1465) and by 31 nt (1991–2136) was observed.

### Comparison of *G. oxydans* 23S rRNA fragmentation with other 23S rRNAs

Fragmentation of 23S rRNA and the presence of intervening sequences (IVSs) in 23S rRNAs were found in several bacteria [39]. We compared the 23S rRNAs GOX1319 and GOX1159 of *G. oxydans* with selected 23S rRNA sequences from such bacteria and also included *E. coli* where the 23S rRNA is not fragmented and IVSs are absent (Additional file 1: Figure S2). For *Salmonella typhimurium* [41], *Rhodobacter sphaeroides*, *Bradyrhizobium japonicum*, *Rhodopseudomonas palustris* [40, 42], *Rhizobium leguminosarum*, and *Agrobacterium radiobacter* [43], it was shown that fragmentation of 23S rRNA occurs by RNA cleavage to remove IVSs. For example, according to the literature IVSs can be found at the positions 131–168, 543–550, and 1176 relative to the *E. coli* sequence (Additional file 1: Figure S2). Close to the first very low coverage region in the mapping for *G. oxydans* an IVS is present in *S. typhimurium* and *R. sphaeroides*. These regions only partially overlap with the relevant *G. oxydans* region and the sequence similarity is low (Additional file 1: Figure S2). For all bacteria shown here and independent of fragmentation, the sequences are quite diverse in this region. For *A. radiobacter* and *R. leguminosarum* fragmentation without the presence of IVS was observed close to position 1500. Close to this region we also observed fragmentation of 23S rRNA in *G. oxydans* (Additional file 1: Figure S2). Sequence similarities among the three bacteria in this region are approximately 70%. The third fragmentation position close to 1750 appears to be present only in *G. oxydans* 23S rRNA (Additional file 1: Figure S2). Sequence similarities between rRNAs with and without fragmentation in this region are quite high, yet there are a few differences in *G. oxydans* compared to the other bacteria. The fourth region with a very low coverage was found only for GOX1159 and not for the other three 23S rRNA copies of *G. oxydans*. In this region GOX1159 differs only at the nucleotide positions 2034 (C for T) and 2119 (the C is absent in GOX1159) compared to the three other 23S rRNAs in *G. oxydans*. This suggests that these positions in the transcript of GOX1159 are specifically relevant for binding and/or cleavage by the processing RNase, likely RNase III as described for others [40, 44]. This fourth fragmentation position is also not known from the other bacteria (Additional file 1: Figure S2). Altogether, no known IVS region described in the literature were found in the 23S rRNAs from *G. oxydans*.

## Discussion

In this study, we estimated the mRNA half-lives in *G. oxydans* 621H on a global scale and detected and analyzed fragmentation of the 23S rRNA transcripts. The mRNA decay plays an important role in the metabolism of nucleic acids in both prokaryotic and eukaryotic cells and also affects fluctuations in protein synthesis and growth [45, 46]. The mRNA abundance in cells is determined by its synthesis and degradation rates. For *E. coli* and the archaeon *S. solfataricus* an inverse relationship between transcript abundance and half-lives was reported [15, 19, 21, 47, 48]. We also observed such an inverse relationship for *G. oxydans*. Many highly abundant transcripts are among the least stable and transcripts with longer half-lives are less abundant. This also supports the view that overall mRNA stability does not play a major role to obtain high mRNA abundance and that a rapid mRNA turnover facilitates fast adaptation to environmental changes. For example, transcripts of highly expressed genes essential for growth can be rapidly degraded in the case of a cell cycle arrest [19]. Nevertheless, as in other studies, not all transcripts follow the inverse relationship between abundance and half-life. For example, the molecular chaperones GroES and GroEL transcripts exhibited high abundance as well as long half-lives in *G. oxydans*. GroES and GroEL are required for proper folding of many proteins [49]. As their activity is required almost independent of environmental conditions, in such cases it is probably advantageous for the cell to support high transcript levels also by long half-lives. Similarly, ATP-proton motive force interconversion is a cellular function that actually needs to be maintained under many conditions. However, in *G. oxydans* this functional category exhibited by far the shortest mRNA half-lives among all categories. With a mean half-life of 3 min the mRNAs of the H^+^-ATP synthase were among the transcripts with the shortest half-lives. The Na^+^-ATP synthase with a mean RNA half-life of 5.1 min exhibited only very low transcript abundance compared to the H+-ATP synthase (80-fold lower). Comparative DNA microarray analysis of the effects of oxygen limitation in *G. oxydans* revealed an approximately 2-fold decreased expression of the H^+^-ATP synthase and 2- to 3-fold increased expression of the Na^+^-ATP synthase [34]. This expression pattern and the abundance of their transcripts suggest that the assumed Na^+^-ATP synthase might play an important role under oxygen limitation and that the H^+^-ATP synthase with the very short mRNA half-lives is the major one under many other conditions. Actually, one would expect that genes of the energy metabolism including ATP synthases are among the medium or most stable transcripts due to their classification as housekeeping genes [50]. Indeed, for *E. coli* the mRNA half-lives of genes associated with energy metabolism (mean 6.3 min) rank in the top 3 categories with the longest half-lives among all groups reported with mean values ranging from 3.8 min to 6.4 min [15]. The *E. coli* ATP synthase transcripts exhibited a mean half-life of 6.2 min in M9 medium, which is twice as long compared to *G. oxydans* (3 min). This 2-fold difference in mRNA half-lives is even more remarkable when also considering the inverse and almost 2-fold difference in the typical doubling time of *E. coli* (~ 60 to 70 min) and *G. oxydans* (~ 100 to 110 min). Thus, the H^+^-dependent ATP synthase could represent a bottleneck in *G. oxydans* under certain conditions, for example in strains engineered for improved growth [13, 14, 51].

The average mRNA half-lives of genes involved in central carbon metabolism did not differ significantly from the global mean (5.7 min). This is attributed to partially high variation of half-lives within functional groups. There is also partially high variation of half-lives associated with the central carbon metabolism in *E. coli* [15], while in *S. solfataricus* transcripts of central metabolic pathways generally showed shorter half-lives [19]. In *S. cerevisiae* transcripts encoding the enzymes that participate in central metabolism are characteristically among those with the longest half-lives [48]. Thus, a general trend is not obvious and the mRNA half-lives in the central metabolism appear to be a species-specific characteristic.

Another species-specific characteristic is fragmentation of 23S rRNA and the absence or presence of segments termed intervening sequence (IVS). While this phenomenon is already known and studied in several bacteria including α-proteobacteria [39, 40], it was not yet reported for *Gluconobacter*. In the γ-proteobacterium *S. typhimurium* fragmentation occurs by RNase III-dependent excision of IVS elements [41, 52]. In α-proteobacteria, for example *Rhizobiaceae*, *Bradyrhizobiaceae*, and *Rhodobacteraceae*, processing of an IVS located close to the 5′-end of the 23S rRNA likely also involves RNase III in an early step. The multistep process generates a fragment of approximately 130 nt from the 5′-end, which is separated by the IVS from the main segment of the 23S rRNA [40, 42, 43]. According to our mapping coverage analysis and sequence comparisons with other 23S rRNAs, no IVS element close to the 5′-end were found, whose excision could possibly lead to a 130 nt fragment. Three potential fragmentation regions were found in all four 23S rRNAs and a fourth fragmentation position only in the 23S rRNA transcript of GOX1159. The potential fragmentation region at nt 1456–1465 is within the same fragmentation region of 23S rRNA from *A. radiobacter* and *R. leguminosarum*. For the other three fragmentation regions no fragmentation was reported in the 23S rRNAs of other α-proteobacteria for which fragmentations at other sites were reported. One of the three is found at nt 1139–1160 within a region without conservation, where also one IVS is present in the 23S rRNAs of *S. typhimurium* and *R. sphaeroides*. The sequences of this region exhibit no or only partial similarity to the region in *G. oxydans*. The third fragmentation region at nt 1716–1770 is in a highly conserved region according to the alignment, yet a fragmentation in other bacteria was not reported. This may be attributed to single nucleotide differences in this conserved region that can enable or disable cleavage by RNase activity, as can be seen at the fourth fragmentation position found only in GOX1159. In GOX1159 this region contains two specific single nucleotide variations compared to the other three 23S rRNA sequences from *G. oxydans*, which may be responsible for recognition and cleavage by RNase activity. Since these variations are located within the fragmentation region and not at their ends or in the flanking regions, the results suggest further rRNA processing of the fragments after the cleavage. Indeed, such secondary maturation steps which further process 23S rRNA 5′- and 3′- ends after RNase III cleavage were already suggested for α-proteobacteria [40].

Generally, fragmentation of 23S rRNA appeared to be phenotypically silent and processing of IVS elements was not required for the production of functional ribosomes [52]. Due to missing phenotypes there is still no convincing explanation for the physiological role of 23S rRNA fragmentation. For *Yersinia enterocolitica* and *S. typhimurium* it was suggested that removing the IVS elements may protect the resulting 23S rRNA fragments from unknown bacteriocins in the gut [53]. For *S. typhimurium* it was also suggested that fragmentation may provide an advantage in stationary phase to quickly adapt to the rapidly changing growth environment experienced in animal hosts [54]. A more general role for rRNA fragmentation was speculated recently in a study of the mammal naked mole-rat in the context of translational fidelity [55]. Naked mole-rat fibroblasts exhibited significantly increased translational fidelity while having comparable translation rates with mouse fibroblasts. In the naked mole-rat the 28S rRNA is also fragmented while in mouse not. It was speculated that rRNA fragmentation may change the folding or dynamics of the large ribosomal subunit, altering the rate of GTP hydrolysis and/or interaction of the large subunit with tRNA during accommodation, thus finally affecting the fidelity of protein synthesis. It will be interesting to see if the biological function of IVS elements is indeed related to translational fidelity of ribosomes by fragmentation of 23S or 28S rRNAs.

## Conclusion

In conclusion, the mRNA decay data from *G. oxydans* showed many similarities to the results obtained in other bacteria and also exhibited some specific differences. Overall, the slightly inverse relationship of transcript abundance and mRNA stability supports the view that mRNA stability does not play a general role to obtain high mRNA abundance. Rather, a quick mRNA turnover for fast adaptation of the cell to environmental changes is made possible by the shorter half-lives of highly expressed genes. The very short mRNA half-lives of the H^+^-ATP synthase, which is likely responsible for the ATP-proton motive force interconversion in *G. oxydans* under many or most conditions according to expression data, is notably in contrast to mRNA decay data from other bacteria. Together with the short mRNA half-lives and the relatively low expression of some genes of the incomplete TCA cycle, which exhibited the lowest expression values in the central carbon metabolism, these could be bottlenecks in *G. oxydans* at some point. This should be considered in future metabolic engineering approaches to further improve growth and biomass yield of *G. oxydans*. The consequences of 23S rRNA fragmentation on growth and fitness of *G. oxydans* are unknown. Our Illumina sequencing and mapping analysis of the RNA fragments isolated from enriched ribosomes revealed three potential fragmentation regions which were previously not known from 23S rRNA fragmentation studies in other bacteria. Further studies are needed to unravel the multistep process of fragmentation in the 23S rRNAs of *G. oxydans*. Although fragmentation of 23S rRNAs appears to be phenotypically silent, it could be relevant under some conditions or for some aspect, for example the fidelity during protein synthesis.

## Additional file

Additional file 1: **Figure S1.** Chromatogram of ribosome enrichment and rRNA obtained. **Figure S2; 23S** rRNA sequence alignment. **Table S1.** mRNA decay data. **Table S2.** FPKM expression values and mRNA half-lives. **Table S3.** mRNA half-lives of operons and monocistronic transcripts. **Table S4.** Amounts of protein and RNA in peak P1, P2, P3, P4. **Table S5.** proteins identified in chromatographic elution fractions. **Table S6.** ribosomal proteins with # tryptic peptides in P1, P2, P3, P4. **Tables S7-S14.** rRNA mapping coverage in exponential phase. **Tables S15-S22.** rRNA mapping coverage in early stationary phase. (ZIP 220 kb)

## Abbreviations

PPP: Pentose phosphate pathway
TCA: Tricarboxylic acid
EDP: Entner-Doudoroff pathway
mDH: Membrane-bound dehydrogenase
IVS: Intervening sequence;
