# Supplementary material for: Global mRNA decay and 23S rRNA fragmentation in Gluconobacter oxydans 621H
Source: BMC Genomics. 2018 Oct 16;19:753. doi: 10.1186/s12864-018-5111-1 (PMC6191907; doi:10.1186/s12864-018-5111-1)
Supplement: Supplementary file 1 — Figure S1. Chromatogram of ribosome enrichment and rRNA obtained. Figure S2; 23S rRNA sequence alignment. Table S1. mRNA decay data. Table S2. FPKM expression values and mRNA half-lives. Table S3. mRNA half-lives of operons and monocistronic transcripts. Table S4. Amounts of protein and RNA in peak P1, P2, P3, P4. Table S5. proteins identified in chromatographic elution fractions. Table S6. ribosomal proteins with # tryptic peptides in P1, P2, P3, P4. Tables S7-S14. rRNA mapping coverage in exponential phase. Tables S15-S22. rRNA mapping coverage in early stationary phase. (ZIP 220 kb) [file 12864_2018_5111_MOESM1_ESM.zip › Additional_Files/Additional_File_1__Figure_S1.docx]

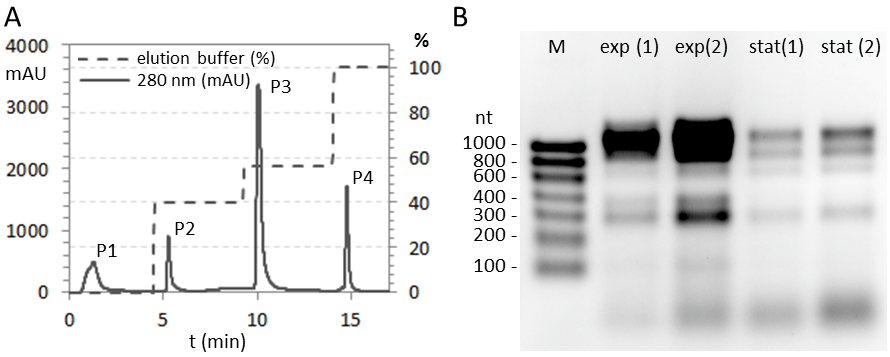


**Figure S1** Chromatogram of the ribosome enrichment (A) and formaldehyde agarose gel analysis of RNA obtained from ribosomes in fraction P3 (B).

A) Aliquots of *G. oxydans* cell-free extracts were loaded onto the two monolithic disks and ribosomes were isolated using a stepwise increase of the % elution buffer (40%, 56%, 100%). During elution the online chromatogram was visually inspected for upcoming peaks according to the absorbance at 280 nm (mAU) to manually collect from the start to the end of a peak into one elution fraction. The four peaks indicating the elution of protein fractions are labeled by P1, P2, P3, and P4.

B) RNA was isolated from peak P3 (A) containing enriched ribosomes from cells in the exponential growth phase (exp) or from the early stationary phase (stat), each in two independent biological replicates (1, 2). M, RiboRuler low range ladder. Gels were loaded with 1.5 µg RNA for exp (1), stat (1) as well as stat (2) and with 3 µg of RNA for exp (2).
