# Supplementary material for: Global mRNA decay and 23S rRNA fragmentation in Gluconobacter oxydans 621H
Source: BMC Genomics. 2018 Oct 16;19:753. doi: 10.1186/s12864-018-5111-1 (PMC6191907; doi:10.1186/s12864-018-5111-1)
Supplement: Supplementary file 1 — Figure S1. Chromatogram of ribosome enrichment and rRNA obtained. Figure S2; 23S rRNA sequence alignment. Table S1. mRNA decay data. Table S2. FPKM expression values and mRNA half-lives. Table S3. mRNA half-lives of operons and monocistronic transcripts. Table S4. Amounts of protein and RNA in peak P1, P2, P3, P4. Table S5. proteins identified in chromatographic elution fractions. Table S6. ribosomal proteins with # tryptic peptides in P1, P2, P3, P4. Tables S7-S14. rRNA mapping coverage in exponential phase. Tables S15-S22. rRNA mapping coverage in early stationary phase. (ZIP 220 kb) [file 12864_2018_5111_MOESM1_ESM.zip › Additional_Files/Additional_File_2__Figure_S2.docx]

Ec_rrlA GGTTAAGCGACT------AA-GCGTACACGGTGGATGCCCTGGCAGTCAGAGGCGATGAA 53

St_rrnH -CTAAGCGTACACGGTGGAT-GCCCTGGCAGTCAGAGGCGATGAAGGGCGTGCTAATCTG 58

Rs_RSP_4295 --------TCAAGCGCGAAAAGGGCGTTTGGTGGATGCCTAGGCAGCAAGAGGCGATGAA 52

Go_GOX1319 ----------------GAGAAGGGCGTTCGGTGGATGCCTTGGCACTAAGAGGCGATGAA 44

Go_GOX1159 ----------------GAGAAGGGCGTTCGGTGGATGCCTTGGCACTAAGAGGCGATGAA 44

Ar_ARAD_RS00940 ------GATTAAGTGTTATAAGGGCATTTAGTGGATGCCTAGGCATGCACAGGCGATGAA 54

Rl_RLEG3_RS21775 ------GATTAAGTGTCGTAAGGGCATTTGGTGGATGCCTTGGCATGCACAGGCGATGAA 54

Rp_RpaI_R0046 ------AATCAAGTGCCTTAAGGGTGTTCGACGGATGCCTTGGCGCTGAGAGGCGATGAA 54

Bj_BJ6T_RS07380 -----CAATCAAGTGCCTTAAGGGTGTTCGGTGGATGCCTTGGCGCTGAGAGGCGATGAA 55

* * * * * **

Ec_rrlA GGACGTGCTAATCTGCGATAAGCGTCGGTAAGGTGATATGAACCGTTATAACCGGCGATT 113

St_rrnH CGATAA---------------GCGCCGGTAAGGTGATATGAACCGTTATAACCGGCGATA 103

Rs_RSP_4295 GGACGTGATACCCTGCGTTAAGCCATGGGGAGCCGGGAATGGGCTTTGATCCA-TGGATG 111

Go_GOX1319 GGACGTGGTACGCTGCGAAAAGCCATGGGGAGCCGCGAACAGGCTTTGATCCG-TGGATG 103

Go_GOX1159 GGACGTGGTACGCTGCGAAAAGCCATGGGGAGCCGCGAACAGGCTTTGATCCG-TGGATG 103

Ar_ARAD_RS00940 GGACGTGATACGCTGCGAAAAGCCGTGGGGAGCTGCGAATAAGCTTTGATCCA-TGGATC 113

Rl_RLEG3_RS21775 GGACGTGATACGCTGCGAAAAGCCGTGGGGAGCTGCGAATGAGCTTTGATCCA-TGGATC 113

Rp_RpaI_R0046 GGACGTGCTACGCTGCGATAAGCCATGGGGAGCTGCGAAGAAGCTTTGATCCG-TGGATT 113

Bj_BJ6T_RS07380 GGACGTGCTACGCTGCGATAAGCCGTGGGGAGCTGCGAAGAAGCTTTGATCCA-TGGATT 114

** ** ** ** * * * ** * ***

Ec_rrlA TCCGAATGGGGAAACCCA------------------------------------------ 131

St_rrnH CCCGAATGGGGAAACCCA------------------------------------------ 121

Rs_RSP_4295 TCCGAATGGGGAAACCCACCTGACATTCTGCTATTGTTATCCAACGGATATCG------- 164

Go_GOX1319 TCCGAATGGGGCAACCCCCTCGCAAGA--------------------------------- 130

Go_GOX1159 TCCGAATGGGGCAACCCCCTCGCAAGA--------------------------------- 130

Ar_ARAD_RS00940 TCCGAATGGGGCAACCCACCTTAAATGCTTAGAAAATCCAAACTGCTGCATAGCA----- 168

Rl_RLEG3_RS21775 TCCGAATGGGGCAACCCACCTTAAATGCTTGGAAAATCATTCTGGTTGTCTGGCCTT--- 170

Rp_RpaI_R0046 TCCGAATGGGGAAACCCACCTTCGATAGCCGGAACTCCAAGGCCTTGCGTTTCGA----- 168

Bj_BJ6T_RS07380 TCCGAATGGGGAAACCCACCTTCGATAGCCGGAACTCCAAGACCTTTGTCGAAAGACATC 174

********** *****

Ec_rrlA ------------------------------------------------------------ 131

St_rrnH ------------------------------------------------------------ 121

Rs_RSP_4295 ------------------------------------------------------------ 164

Go_GOX1319 ------------------------------------------------------------ 130

Go_GOX1159 ------------------------------------------------------------ 130

Ar_ARAD_RS00940 -----------------------------------------------------------G 169

Rl_RLEG3_RS21775 ----------------------------------------------TGGCCCGGCAACCC 184

Rp_RpaI_R0046 ----------------------------------------------AAGAGACGTGAGGG 182

Bj_BJ6T_RS07380 GGTGTGGGGTTCGATCAGATGATGTGAGAAGCCAGGCCTTTAGATTTCGATCGAAGAGGT 234

Ec_rrlA -------------GTGTGTTTCGACACACTATCATTAACTGAATCCATAGGTTAATGAGG 178

St_rrnH -------------GTGTGATTCGTCACACTATCATTAACTGAATCCATAGGTTAATGAGG 168

Rs_RSP_4295 ---------ATAGTGGGGTGAGACAGGTATCTTAACCCTGAATACATAGGGGTTTTGAAG 215

Go_GOX1319 ----------------------------GGATCATGCACTGAATACATAGGTGTATGAGG 162

Go_GOX1159 ----------------------------GGATCATGCACTGAATACATAGGTGTATGAGG 162

Ar_ARAD_RS00940 CTTGGGTTTCTAAGCATTGTGATAAGGTATCTACACCTGAATAAAATAGGGTGTAAGAAG 229

Rl_RLEG3_RS21775 GAGTGGTTTCCAAGCATTGTGATAAGGTATCTACACCTGAAT-ACATAGGGTGTAAGAAG 243

Rp_RpaI_R0046 TTTGGATTTCCGGTTATCAAGAGAAGGTATGAGATCTCTGAATACATAGGAGGTTTCAAG 242

Bj_BJ6T_RS07380 TTTGGATTTCCGGTTATCAAGAGAAGGTATGAGACTTCTGAATACATAGGAGGTTTCAAG 294

* * *

Ec_rrlA CGAACCGGGGGAACTGAAACATCTAAGTACCCCGAGGAAAAGAAATCAACCGAGATTCCC 238

St_rrnH CGAACCGGGGGAACTGAAACATCTAAGTACCCCGAGGAAAAGAAATCAACCGAGATTCCC 228

Rs_RSP_4295 CGAACCCGGGGAACTGAAACATCTAAGTACCCGGAGGAAAGGAAATCAACAGAGACTCCG 275

Go_GOX1319 CAAACCCGGGGAACTGAAACATCTCAGTACCTGGAGGAAAAGACATCAACAGAGATTCCG 222

Go_GOX1159 CAAACCCGGGGAACTGAAACATCTCAGTACCTGGAGGAAAAGACATCAACAGAGATTCCG 222

Ar_ARAD_RS00940 CGAACGCAGGGAACTGAAACATCTAAGTACCTGCAGGAAAGGACATCAACCGAGACTCCG 289

Rl_RLEG3_RS21775 CGAACGCAGGGAACTGAAACATCTAAGTACCTGCAGGAAAGGACATCAACCGAGACTCCG 303

Rp_RpaI_R0046 CGAACCCAGGGAACTGAAACATCTAAGTACCTGGAGGAAAGGACATCAACCGAGACTCCG 302

Bj_BJ6T_RS07380 CAAACCCAGGGAACTGAAACATCTAAGTACCTGGAGGAAAGGACATCAACAGAGACTCCG 354

* *** **************** ****** ****** ** ****** **** ***

Ec_rrlA CCAGTAGCGGCGAGCGAACGGGGAGCAGCCCAGAGCCTGAATCAGTGTGTGTGTTAGTGG 298

St_rrnH CCAGTAGCGGCGAGCGAACGGGGAGGAGCCCAGAGCCTGAATCAGCATGTGTGTTAGTGG 288

Rs_RSP_4295 CTAGTAGTGGCGAGCGAACGCGGACCAGCCGATCTCCGAAGAGTG----------ACTGG 325

Go_GOX1319 CTAGTAGTGGCGAGCGAACGCGGAGCAGGCCAATGCCTGATCAGGAAGA-----AGCTGA 277

Go_GOX1159 CTAGTAGTGGCGAGCGAACGCGGAGCAGGCCAATGCCTGATCAGGAAGA-----AGCTGA 277

Ar_ARAD_RS00940 CAAGTAGTGGCGAGCGAACGCGGACCAGGCCAGTGGCAATGAGTGTTAA-----AGTGGA 344

Rl_RLEG3_RS21775 CAAGTAGTGGCGAGCGAACGCGGACCAGGCCAGTGGCAATTGTGATTAA-----AGTGGA 358

Rp_RpaI_R0046 CTAGTAGTGGCGAGCGAACGCGGACCAGGCCAGTCATCATTGGAAGACA-----ATTGGA 357

Bj_BJ6T_RS07380 TTAGTAGTGGCGAGCGAACGCGGACCAGGCCAGTGATACATCAAAGACA-----ATCGGA 409

***** ************ *** ** * * *

Ec_rrlA AAGCGTCTGGAAAGGCGTGCGATACAGGGTGACAGCCCCGTACACAAAAATGCACATGCT 358

St_rrnH AAGCGTCTGGAAAGGCGCGCGATACAGGGTGACAGCCCCGTACACAAAAGCGCATGTGCT 348

Rs_RSP_4295 AATGGCCTGGAAAGGCCAGCCACAGCGGGTGACAGCCCCGTACAGGAAG------CTCCA 379

Go_GOX1319 -ACGGTCTGGAAAGTCCGGCAAGAATGGGTGATAGCCCCGTAAGCGTAG-----TGTTCT 331

Go_GOX1159 -ACGGTCTGGAAAGTCCGGCAAGAATGGGTGATAGCCCCGTAAGCGTAG-----TGTTCT 331

Ar_ARAD_RS00940 -AGAACCTGGAAAGGTTTGCCGTAGAGGGTGATAGCCCCGTACGCGTAG-----ATACAC 398

Rl_RLEG3_RS21775 -ACGCTCTGGAAAGTGCGGCCGTAGTGGGTGACAGCCCCGTACGCGTAG-----ATATCA 412

Rp_RpaI_R0046 ATCTGTCAGGAAAGCAGAGCCTTAGAGGGTGATAGCCCCGTACAAGTAA-----TTCAAC 412

Bj_BJ6T_RS07380 ACCGGTCAGGAAAGCCGGGCCTCAGAGGGTGATAGCCCCGTACGAGTAA-----TGCGAT 464

* ****** ** * ****** ********* *

Ec_rrlA GTGAGCTCGATGAGTAGGGCGGGACACGTGGTATCCTGTCTGAATATGGGGGGACCATCC 418

St_rrnH GTGAGCTCGATGAGTAGGGCGGGACACGTGGTATCCTGTCTGAATATGGGGGGACCATCC 408

Rs_RSP_4295 GGAGACATATCAAGTAGGGCGGGACACGTGAAATCCTGTCTGAAGATCGGGGGACCACCC 439

Go_GOX1319 GATGAGGATTCGAGTAGGGCGGGGCACGTGAAACCCTGTCTGAACATGGGGGGACCACCC 391

Go_GOX1159 GATGAGGATTCGAGTAGGGCGGGGCACGTGAAACCCTGTCTGAACATGGGGGGACCACCC 391

Ar_ARAD_RS00940 TTATTGTCCTAGAGTAGGGCGGGACACGTGAAATCCTGTCTGAACATGGGGCGACCACGC 458

Rl_RLEG3_RS21775 TGATTGTCCTAGAGTAGGGCGGGACACGAGAAATCCTGTCTGAACATGGGGAGACCACTC 472

Rp_RpaI_R0046 CATTGATGCTCGAGTAAGGCGGGACACGTGAAATCCTGTCTGAACATGGGGGGACCACCC 472

Bj_BJ6T_RS07380 GATGTATCCACGAGTAAGGCGGGACACGTGAAATCCTGTCTGAACGCGG-GGGACCACCC 523

**** ****** **** * * ********** * * ***** *

Ec_rrlA TCCAAGGCTAAATACTCCTGACTGACCGATAGTGAACCAGTACCGTGAGGGAAAGGCGAA 478

St_rrnH TCCAAGGCTAAATACTCCTGACTGACCGATAGTGAACCAGTACCGTGAGGGAAAGGCGAA 468

Rs_RSP_4295 CCGAAGGCTAAGTACTCCTTGCTGACCGATAGCGAACCAGTACCGTGAGGGAAAGGTGAA 499

Go_GOX1319 TCCAAGCCTAAATACTCCTTAGTGACCGATAGCGAACAAGTACCGTGAGGGAAAGGTGAA 451

Go_GOX1159 TCCAAGCCTAAATACTCCTTAGTGACCGATAGCGAACAAGTACCGTGAGGGAAAGGTGAA 451

Ar_ARAD_RS00940 TCCAAGCCTAAGTACTCGTGCATGACCGATAGCGAACCAGTACCGTGAGGGAAAGGTGAA 518

Rl_RLEG3_RS21775 TCCAAGCCTAAGTACTCGTGCATGACCGATAGCGAACAAGTACCGTGAGGGAAAGGTGAA 532

Rp_RpaI_R0046 TCCAAGCCTAAGTACTCCTCAGCGACCGATAGTGAACCAGTACCGTGAGGGAAAGGTGAA 532

Bj_BJ6T_RS07380 TCCAAGCCTAAGTACTCCTCAGCGACCGATAGTGAACCAGTACCGTGAGGGAAAGGTGAA 583

* *** **** ***** * ********* **** ****************** ***

Ec_rrlA AAGAACCCCGGCGAGGGGAGTGAAAAAGAACCTGAAACCGTGTACGTACAAGCAGTGGGA 538

St_rrnH AAGAACCCCGGCGAGGGGAGTGAAAAAGAACCTGAAACCGTGTACGTACAAGCAGTGGGA 528

Rs_RSP_4295 AAGCACCCCGACGAGGGGAGTGAAACAGTACCTGAAACCGGACGCCTACAAGCAGTCGGA 559

Go_GOX1319 AAGCACCCCGATGAGGGGAGTGAAAGAGAC-CTGAAACCGGACGCCTACAAGCAGTCG-- 508

Go_GOX1159 AAGCACCCCGATGAGGGGAGTGAAAGAGAC-CTGAAACCGGACGCCTACAAGCAGTCG-- 508

Ar_ARAD_RS00940 AAGCACCCCGACAAGGGGAGTGAAATAGAACCTGAAACTGGATGCCTACAAACAGTCGGA 578

Rl_RLEG3_RS21775 AAGCACCCCGACAAGGGGAGTGAAATAGAACCTGAAACCGGATGCCTACAAACAGTCGGA 592

Rp_RpaI_R0046 AAGCACCCCGACGAGGGGAGTGAAATAGTTCCTGAAATCGGACACCTACAAACAGACGGA 592

Bj_BJ6T_RS07380 AAGCACCCCGACGAGGGGAGTGAAATAGA-CCTGAAACCGGACACCTACAAACAGATGGA 642

*** ****** ************ ** ****** * * ***** *** *

Ec_rrlA GCACG------------------------------------------------------- 543

St_rrnH GCCCCACCACTAAGCCAGTGGTGAACTCCACATCCGCATCCTTTGCTGAGGATACGGTTA 588

Rs_RSP_4295 GGG-----T--------------------------------------------------- 563

Go_GOX1319 ----GAGCC--------------------------------------------------- 513

Go_GOX1159 ----GAGCC--------------------------------------------------- 513

Ar_ARAD_RS00940 GCCCG------------------------------------------------------- 583

Rl_RLEG3_RS21775 GCCCG------------------------------------------------------- 597

Rp_RpaI_R0046 GCCCAAGAT--------------------------------------------------- 601

Bj_BJ6T_RS07380 GCCCAAGAT--------------------------------------------------- 651

Ec_rrlA ----------------------------------------------CTTAGGCGTGTGAC 557

St_rrnH ACGGAGCGAAAGCGACGTTCAACCGCAAAACAAGCAGAGGGGGCTTAGTGGTGGGGTGAC 648

Rs_RSP_4295 ----------------------------------------------CCATGAGACCTGAC 577

Go_GOX1319 ----------------------------------------------TCTTATGGGGTGAC 527

Go_GOX1159 ----------------------------------------------TCTTATGGGGTGAC 527

Ar_ARAD_RS00940 --------------------------------------------------CAAGGGTGAC 593

Rl_RLEG3_RS21775 --------------------------------------------------CAAGGGTGAC 607

Rp_RpaI_R0046 ----------------------------------------------ACGTTCTGGGTGAC 615

Bj_BJ6T_RS07380 ----------------------------------------------ACGTTCTGGGTGAC 665

****

Ec_rrlA TGCGTACCTTTTGTATAATGGGTCAGCGACTTATATTCTGTAGCAAGGTTAACCGAATAG 617

St_rrnH TGCGTACCTTTTGTATAATGGGTCAGCGACTTATATTCTGTAGCAAGGTTAACCGAATAG 708

Rs_RSP_4295 GGCGTACCTTTTGTATAATGGGTCAACGACTTGGTCTCACGAGCAAGCTTAAGCCGGTAG 637

Go_GOX1319 GGCGTACCTTTTGTATAATGGGTCAGCGAGTTTCTGTTTGCAGCGAGCTTAAGCCGTTAG 587

Go_GOX1159 GGCGTACCTTTTGTATAATGGGTCAGCGAGTTTCTGTTTGCAGCGAGCTTAAGCCGTTAG 587

Ar_ARAD_RS00940 GGCGTACCTTTTGTATAATGGGTCAACGACTTAGTGTAACAAGCAAGCTTAAGCCGGTAG 653

Rl_RLEG3_RS21775 GGCGTACCTTTTGTATAATGGGTCAACGACTTAGTGTAACAAGCAAGCTTAAGCCGGTAG 667

Rp_RpaI_R0046 GTCGTACCTTTTGTATTATGGGCCAGCGACTTAATTTAACGAGCAAGCTTAAGCCGGTAG 675

Bj_BJ6T_RS07380 ATCGTACCTTTTGTATTATGGGCCAGCGACTTAATTTAACGAGCAAGCTTAAGCCGATAG 725

************** ***** ** *** ** * *** ** **** * ***

Ec_rrlA -GGGAGCCGAAGGGAAACCGAGTCTTAACTGGGCGT-TAAGTTGCAGGGTATAGACCCGA 675

St_rrnH -GGGAGCCGGAGGGAAACCGAGTCTTAATTGGGCGT-TAAGTTGCAGGGTATAGACCCGA 766

Rs_RSP_4295 GTGGAGGCGCAGCGAAAGCGAGTCTTAAAAGGGCGT-CGAGTTCGTGGGATCAGACCCGA 696

Go_GOX1319 GTGTAGGCGTAGCGAAAGCGAGTCTGAATAGGGCGAATGAGTTGCTGGCAGAAGACCCGA 647

Go_GOX1159 GTGTAGGCGTAGCGAAAGCGAGTCTGAATAGGGCGAATGAGTTGCTGGCAGAAGACCCGA 647

Ar_ARAD_RS00940 GTGTAGGCGAAGCGAAAGCGAGTCTGAATAGGGCGATTTAGTTTGTTGCATTAGACCCGA 713

Rl_RLEG3_RS21775 GTGTAGGCGAAGCGAAAGCGAGTCTGAATAGGGCGATATAGTTTGTTGCATTAGACCCGA 727

Rp_RpaI_R0046 GTGTAGGCGCAGCGAAAGCGAGTCTGAATAGGGCGT-CAAGTTCGTTGGATTAGACCCGA 734

Bj_BJ6T_RS07380 GCGAAGGCGCAGCGAAAGCGAGTCTGAATAGGGCGT-CAAGTTCGTTGTATTAGACCCGA 784

* ** ** ** **** ******* ** ***** **** * ********

Ec_rrlA AACCCGGTGATCTAGCCATGGGCAGGTTGAAGGTTGGGTAACACTAACTGGAGGACCGAA 735

St_rrnH AACCCGGTGATCTAGCCATGGGCAGGTTGAAGGTTGGGTAACACTAACTGGAGGACCGAA 826

Rs_RSP_4295 AACCAGGTGATCTAGCCATGAGCAGGATGAAGTCAGGGTAACACCTGATGGAGGTCCGAA 756

Go_GOX1319 AACCGAGTGATCTAGCCATGGCCAGGCTGAAGGTGCGGTAACACGCACTGGAGGGCCGAA 707

Go_GOX1159 AACCGAGTGATCTAGCCATGGCCAGGCTGAAGGTGCGGTAACACGCACTGGAGGGCCGAA 707

Ar_ARAD_RS00940 AACCGAGTGATCTAGCCATGAGCAGGTTGAAGGTTGGGTAACACCAACTGGAGGACCGAA 773

Rl_RLEG3_RS21775 AACCGAGTGATCTAGCCATGAGCAGGTTGAAGGTTGGGTAACACCAACTGGAGGACCGAA 787

Rp_RpaI_R0046 AACCTAGTGATCTAGCCATGAGCAGGTTGAAGGTGAGGTAACACTCACTGGAGGACCGAA 794

Bj_BJ6T_RS07380 AACCTAGTGATCTAGCCATGAGCAGGTTGAAGGTGAGGTAACACTCACTGGAGGACCGAA 844

**** ************** **** ***** ******** ****** *****

Ec_rrlA CCGACTAATGTTGAAAAATTAGCGGATGACTTGTGGCTGGGGGTGAAAGGCCAATCAAAC 795

St_rrnH CCGACTAATGTTGAAAAATTAGCGGATGACCTGTGGCTGGGGGTGAAAGGCCAATCAAAC 886

Rs_RSP_4295 CCAACACCCGTTGAAAAGGGTCTGGATGACTTGTGGCTAGGGGTGAAAGGCCAATCAAAC 816

Go_GOX1319 CCCACGCCTGTTGAAAAAGTCGGGGATGAGCTGTGGCTAGGGGTGAAAGGCCAATCAAAC 767

Go_GOX1159 CCCACGCCTGTTGAAAAAGTCGGGGATGAGCTGTGGCTAGGGGTGAAAGGCCAATCAAAC 767

Ar_ARAD_RS00940 CCCGCATCTGTTGCAATAGATTGGGATGACTTGTGGCTAGGGGTGAAAGGCCAATCAAAC 833

Rl_RLEG3_RS21775 CCCGCATCTGTTGCAATAGATTGGGATGACTTGTGGCTAGGGGTGAAAGGCCAATCAAAC 847

Rp_RpaI_R0046 CGGGTGCCTGTTGAAAAAGGCTCCGATGACTTGTGGTTAGGGGTGAAAGGCCAATCAAAC 854

Bj_BJ6T_RS07380 CGGGTGCCTGTTGAAAAAGGCTCCGATGACTTGTGGTTAGGGGTGAAAGGCCAATCAAAC 904

* **** ** ***** ***** * *********************

Ec_rrlA CGGGAGATAGCTGGTTCTCCCCGAAAGCTATTTAGGTAGCGCCTCGTGAATTCATCTCCG 855

St_rrnH CGGGAGATAGCTGGTTCTCCCCGAAAGCTATTTAGGTAGCGCCTCGTGAATTCATCTCCG 946

Rs_RSP_4295 CTGGAGATAGCTGGTTCTCCGCGAAAGCTATTTAGGTAGCGCCTCGGACGAATACCTCGG 876

Go_GOX1319 TCGGAGATAGCTGGTTCTCCGCGAAATCTATTGAGGTAGATCGTCGGGTGTTTACCCTGG 827

Go_GOX1159 TCGGAGATAGCTGGTTCTCCGCGAAATCTATTGAGGTAGATCGTCGGGTGTTTACCCTGG 827

Ar_ARAD_RS00940 TCGGAAATAGCTGGTTCTCCGCGAAATCTATTTAGGTAGAGCGTCGAGCGAATACTCCCG 893

Rl_RLEG3_RS21775 TCGGAAATAGCTGGTTCTCCGCGAAATCTATTTAGGTAGAGCGTCGAGCGAATACCCCCG 907

Rp_RpaI_R0046 TGGGAAATAGCTGGTTCTCCGCGAAAGATATTTAGGTATCGCCTCGGACGAATACCTCAG 914

Bj_BJ6T_RS07380 TGGGAAATAGCTGGTTCTCCGCGAAAGATATTTAGGTATCGCCTCGGATGAATACCTCAG 964

*** ************** ***** **** ***** * *** * *

Ec_rrlA GGGGTAGAGCACTGTTTCGGCAAGGGGGTCATCCCGACTTACCAACCCGATGCAAACTGC 915

St_rrnH GGGGTAGAGCACTGTTTCGGCTAGGGGGCCATCCCGGCTTACCAACCCGATGCAAACTGC 1006

Rs_RSP_4295 GGGGTAGAGCACTGCATGGATGATGGGGGCCCACAGCCTTACTGAGTCTAAGCAAACTCC 936

Go_GOX1319 GGGGTAGAGCACTGGATGGGCTAGGGGGGCCCAAAGCCTTACCAAACCTAACCAAACTCC 887

Go_GOX1159 GGGGTAGAGCACTGGATGGGCTAGGGGGGCCCAAAGCCTTACCAAACCTAACCAAACTCC 887

Ar_ARAD_RS00940 GGGGTAGAGCACTGGATGGGCTATGGGGACTCACCGTCTTACTGATCCTAACCAAACTCC 953

Rl_RLEG3_RS21775 GGGGTAGAGCACTGGATGGGCTATGGGGACTCACCGTCTTACTGATCCTAACCAAACTCC 967

Rp_RpaI_R0046 GGGGTAGAGCACTGGATGGGCTAGGGGGACTTACCGTCTTACCAAACCCAACCAAACTCC 974

Bj_BJ6T_RS07380 GGGGTAGAGCACTGGATGGGCTAGGGGGACTTACCGTCTTACCAAACCCAACCAAACTCC 1024

************** * * * **** * * ***** * * * ****** *

Ec_rrlA GAATACCGGAGAATGTTATCACGGGAGACACACGGCGGGTGCTAACGTCCGTCGTGAAGA 975

St_rrnH GAATACCGGAGAATGTTATCACGGGAGACACACGGCGGGTGCTAACGTCCGTCGTGAAGA 1066

Rs_RSP_4295 GAATACCCGAGAGTACTA-TCCGGGAGACACACGGCGGGTGCTAACGTCCGTCGTGAAGA 995

Go_GOX1319 GAATACCCAGGAGTATAG-CCCGGCAGACAGACAGTGGGTGCTAAGGTC**C**ATTGTCGAGA 946

Go_GOX1159 GAATACCCAGGAGTATAG-CCCGGCAGACAGACAGTGGGTGCTAAGGTC**T**ATTGTCGAGA 946

Ar_ARAD_RS00940 GAATACCGGGAAGTACTA-CTCGGCAGACACACGGCGGGTGCTAACGTCCGTCGTGAAAA 1012

Rl_RLEG3_RS21775 GAATACCGGGGAGTACTA-CTCGGCAGACACACGGCGGGTGCTAACGTCCGTCGTGAAAA 1026

Rp_RpaI_R0046 GAATACCTGAGAGTACTA-TCCGGGAGTCACACGGCGGGTGCTAACGTCCGTCGTGGAGA 1033

Bj_BJ6T_RS07380 GAATACCTGAGAGTACTA-TCCGGGAGTCACACGGCGGGTGCTAACGTCCGTCGTGGAGA 1083

******* * * *** ** ** ** * ********* *** * ** * *

Ec_rrlA GGGAAACAACCCAGACCGCCAGCTAAGGTCCCAAAGTCATGGTTAAGTGGGAAACGATGT 1035

St_rrnH GGGAAACAACCCAGACCGCCAGCTAAGGTCCCAAAGTCATGGTTAAGTGGGAAACGATGT 1126

Rs_RSP_4295 GGGAAACAACCCTGACCTGCAGCTAAGGCCCCCAATTCGTGGCTAAGTGGGAAAGCATGT 1055

Go_GOX1319 GGGAAACAGCCCAGACCACCAGCTAAGGCCCCTAAATCGTGGCTAAGTGGGAAAGGATGT 1006

Go_GOX1159 GGGAAACAGCCCAGACCACCAGCTAAGGCCCCTAAATCGTGGCTAAGTGGGAAAGGATGT 1006

Ar_ARAD_RS00940 GGGCAACAACCCTAACCTCCAGCTAAGGTCCCCAAGTCATGGCTAAGTGGGAAAGGATGT 1072

Rl_RLEG3_RS21775 GGGCAACAACCCTAACCTCCAGCTAAGGTCCCCAAGTCATGGCTAAGTGGGAAAGGATGT 1086

Rp_RpaI_R0046 GGGAAACAACCCTGACCTACAGCTAAGGCCCCCAATTCGTGGCTAAGTGGGAAAGGATGT 1093

Bj_BJ6T_RS07380 GGGAAACAACCCGGACCTACAGCTAAGGCCCCTAATTCGTGGCTAAGTGGGAAAGGATGT 1143

*** **** *** *** ********* *** ** ** *** *********** ****

Ec_rrlA GGGAAGGCCCAGACAGCCAGGATGTTGGCTTAGAAGCAGCCATCATTTAAAGAAAGCGTA 1095

St_rrnH GGGAAGGCCCAGACAGCCAGGATGTTGGCTTAGAAGCAGCCATCATTTAAAGAAAGCGTA 1186

Rs_RSP_4295 GGGACGGCCAAAACAACCAGGAGGTTGGCTTAGAAGCAGCCATCCTTTAAAGATAGCGTA 1115

Go_GOX1319 GGGGATTCCAAAACAACCAGGAGGTTGGCTTAGAAGCAGCCATCCTTTAAAGAAAGCGTA 1066

Go_GOX1159 GGGGATTCCAAAACAACCAGGAGGTTGGCTTAGAAGCAGCCATCCTTTAAAGAAAGCGTA 1066

Ar_ARAD_RS00940 GAGGATCCCAAAACAACCAGGATGTTGGCTTAGAAGCAGCCATCATTTAAAGAAAGCGTA 1132

Rl_RLEG3_RS21775 GAGGATCCCAAAACAACCAGGATGTTGGCTTAGAAGCAGCCATCATTTAAAGAAAGCGTA 1146

Rp_RpaI_R0046 GGAAATCCCAAAACAACCAGGAGGTTGGCTTAGAAGCAGCCATCCTTTAAAGAAAGCGTA 1153

Bj_BJ6T_RS07380 GGAAATCCCAAAACAACCAGGAGGTTGGCTTAGAAGCAGCCATCCTTTAAAGAAAGCGTA 1203

* ** * *** ****** ********************* ******** ******

Ec_rrlA ATAGCTCACTGGTCGAGTCGGCCTGCGC-----GGAAGATGTAACGGGGCTAAACCATGC 1150

St_rrnH ATAGCTCACTGGTCGAGTCGGCCTGCGC-----GGAAGATGTAACGGGGCTAAACCATGC 1241

Rs_RSP_4295 ACAGCTCACTGGTCTAGATAAGCTGTCCTGCGGCGAAGATGTAACGGGGCTCAAGCCACG 1175

Go_GOX1319 ATAGCTCACTGGTCTATTAGAAAC--CCTGCGCCGAAAATGTAACGGGGCTCAAGCCACG 1124

Go_GOX1159 ATAGCTCACTGGTCTATTAGAAAC--CCTGCGCCGAAAATGTAACGGGGCTCAAGCCACG 1124

Ar_ARAD_RS00940 ACAGCTCACTGGTCTAGTCAAGGGTCTTTGCGCCGAAAATGTAACGGGGCTAAAGCCATG 1192

Rl_RLEG3_RS21775 ACAGCTCACTGGTCTAAATAAGGGTCTTTGCGCCGAAAATGTAACGGGGCTGAAGCCATG 1206

Rp_RpaI_R0046 ACAGCTCACTGGTCTAAATAAGGGTTTCTGCGCCGAAGATGTAACGGGGCTCAAGCCACG 1213

Bj_BJ6T_RS07380 ACAGCTCACTGGTCTAAATAAGGGTTTCTGCGCCGAAGATGTAACGGGGCTCAAGCCACG 1263

* ************ * *** ************* ** *

Ec_rrlA A-CCGAAGCTGCGGCAGCGACACTATG--------------------------------- 1176

St_rrnH A-CCGAAGCTGCGGCAGCGAACGTATCACCCAAGACAA--------CTTTACGGAGTT-- 1290

Rs_RSP_4295 AGCCGAAGCTCAGGATGCACAGCAATGTGCGTGGT----AGCGGAGCGTTCCGTGATATA 1231

Go_GOX1319 TGCCGAAGCTGTGGGTGCATAC---TATGTATGCGCGGTAGCGGAGCGTTCCGTAAGT-- 1179

Go_GOX1159 TGCCGAAGCTGTGGGTGCATAC---TATGTATGCGCGGTAGCGGAGCGTTCCGTAAGT-- 1179

Ar_ARAD_RS00940 CACCGAAGCTGAGGATTTGCGAG--CAATCGCAAGTGGTAGCGGAGCGTTCCGTAAGC-- 1248

Rl_RLEG3_RS21775 CACCGAAGCTGAGGATGTGTAGC----AATACACGTGGTAGCGGAGCGTTCCGTAAGC-- 1260

Rp_RpaI_R0046 AGCCGAAGCTTAGGGTGTGCGCA------AGCACGCGGTAGCGGAGCGTTCTGTAAGC-- 1265

Bj_BJ6T_RS07380 AGCCGAAGCTTAGGGTGTGATCCGCAAGGGTCACGCGGTAGCGGAGCGTTCTGTAAGC-- 1321

******** **

Ec_rrlA ------------------------------------------------------------ 1176

St_rrnH -------GACGATTGACGGAGCGAAGCGA---CGTCAAAGCGTTCATTAAAGTCGAGTTG 1340

Rs_RSP_4295 GCTCATTGTGTGTTTATCGAACGCACCACCGGTCCGAACGAGGGCACTGCCCTCAAGTAG 1291

Go_GOX1319 ------------------------------------------------------------ 1179

Go_GOX1159 ------------------------------------------------------------ 1179

Ar_ARAD_RS00940 ------------------------------------------------------------ 1248

Rl_RLEG3_RS21775 ------------------------------------------------------------ 1260

Rp_RpaI_R0046 ------------------------------------------------------------ 1265

Bj_BJ6T_RS07380 ------------------------------------------------------------ 1321

Ec_rrlA ------------TGTTGTTGGGTAGGGGAGCGTTCTGTAAGCCTGTGAAGGTGTGCTGTG 1224

St_rrnH GCTTAGGGATACGTTCGTTGGGTAGGGGAGCGTTCTGTAAGCCTGTGAAGGTGTGTCGTG 1400

Rs_RSP_4295 CGAAGCGGTAGGGCGCGCGGTAGCGCACACAAAGAGCTTTCTGTGAAGCCGGGCCGTAAG 1351

Go_GOX1319 -----------------------------------------CTGCGAAGGAGACGGGGTG 1198

Go_GOX1159 -----------------------------------------CTGCGAAGGAGACGGGGTG 1198

Ar_ARAD_RS00940 -----------------------------------------CTGTGAAGGGGTACCTGTG 1267

Rl_RLEG3_RS21775 -----------------------------------------CGATGAAGGGAGACCCGTG 1279

Rp_RpaI_R0046 -----------------------------------------CTGCGAAGGGCGACCCGTG 1284

Bj_BJ6T_RS07380 -----------------------------------------CTGCGAAGGGCGACTCGTG 1340

*

Ec_rrlA AGGCATGCTGGAGGTATCAGAAGTGCGAATGCTGACATAAGTAACGATAAAGCGGGTGAA 1284

St_rrnH AGGCATGCTGGAGGTATCAGAAGTGCGAATGCTGACATAAGTAACGATAAAGCGGGTGAA 1460

Rs_RSP_4295 GCATCCGGTGGAGAGATCGGAAGCGAGAATGTTGACATGAGTAGCGACAAACAGGGTGAG 1411

Go_GOX1319 ACCCTCTCTGGAGATATCGGAAGTGCGAATGCTGACATGAGTAGCGACAAACAGTGCGAG 1258

Go_GOX1159 ACCCTCTCTGGAGATATCGGAAGTGCGAATGCTGACATGAGTAGCGACAAACAGTGCGAG 1258

Ar_ARAD_RS00940 AGGGGCCCTGGAGGTATCGGAAGTGCGAATGTTGACATGAGTAACGATAAAGAGGGTGAG 1327

Rl_RLEG3_RS21775 AGGGCTCCTGGAGGTATCGGAAGTGCGAATGTTGACATGAGTAACGATAAAGAGGGTGAG 1339

Rp_RpaI_R0046 AGGGCGCCTGGAGGTATCAGAAGTGCGAATGCTGGCATGAGTAACGACAAACACTGTGAA 1344

Bj_BJ6T_RS07380 AGAGCGCCTGGAGGTATCAGAAGTGCGAATGCTGGCATGAGTAACGACAAACACTGTGAA 1400

***** *** **** * ***** ** *** **** *** *** * **

Ec_rrlA AAGCCCGCTCGCCGGAAGACCAAGGGTTCCTGTCCAACGTTAATCGGGGCAGGGTGAGTC 1344

St_rrnH AAGCCCGCTCGCCGGAAGACCAAGGGTTCCTGTCCAACGTTAATCGGGGCAGGGTGAGTC 1520

Rs_RSP_4295 AGACCCTGTCGCCGAAAGTCCAAGGGTTCCTGCTTAAAGCTAATCTGAGCAGGGTAAGCC 1471

Go_GOX1319 AAACACTGTCGCCGAAAGTCCAAGGGTTCCTGCGCAAGGTTAATCCACGCAGGGTGAGCC 1318

Go_GOX1159 AAACACTGTCGCCGAAAGTCCAAGGGTTCCTGCGCAAGGTTAATCCACGCAGGGTGAGCC 1318

Ar_ARAD_RS00940 AGACCCTCTCGCCGAAAGACCAAGGGTTCCTGCTTAAAGTTAATCTGAGCAGGGTTAGCC 1387

Rl_RLEG3_RS21775 AGACCCTCTCGCCGAAAGACCAAGGGTTCCTGCTTAAAGTTAATCTGAGCAGGGTTAGCC 1399

Rp_RpaI_R0046 AGACAGTGTCGCCGAAAGTCCAAGGGTTCCTGCGTAAAGTTAATCTTCGCAGGGTTAGCC 1404

Bj_BJ6T_RS07380 AGACAGTGTCGCCGAAAGTCCAAGGGTTCCTGCGTAAAGTTAATCTTCGCAGGGTTAGCC 1460

* * ****** *** ************* ** * ***** ******* ** *

Ec_rrlA GACCCCTAAGGCGAGGCCGAAAGGCGTAGTCGATGGGAAACAGGTTAATATTCCTGTACT 1404

St_rrnH GACCCCTAAGGCGAGGCCGAAAGGCGTAGTCGATGGGAAACGGGTTAATATTCCCGTACT 1580

Rs_RSP_4295 GGCCCCTAAGGCGAGGCCGAAAGGCGTAGTCGATGGGAACCAGGTTAATATTCCTGGGCC 1531

Go_GOX1319 GGCCCCTAAGGCGAGGGCGAGAGCCGTAGTCGATGGGAACCAGTTCAATATTACTGGGCC 1378

Go_GOX1159 GGCCCCTAAGGCGAGGGCGAGAGCCGTAGTCGATGGGAACCAGTTCAATATTACTGGGCC 1378

Ar_ARAD_RS00940 GGCCCCTAAGACGAGGCGGACACGCGTAGTCGATGGGAACCACGTTAATATTCGTGGGCC 1447

Rl_RLEG3_RS21775 GGCCCCTAAGGCGAGGCAGAAATGCGTAGTCGATGGGAACCACGTTAATATTCGTGGGCC 1459

Rp_RpaI_R0046 GGTCCCTAAGGCGAGGCCGAAAGGCGTAGTCGATGGGAATCACGTGAATATTCGTGAGCC 1464

Bj_BJ6T_RS07380 GGTCCCTAAGGCGAGGCCGAAAGGCGTAGTCGATGGGAATGCAGTGAATATTCTGCAGCC 1520

* ******* ***** ** * *************** * ****** *

Ec_rrlA TGGTGTTACTGCGAAGGGGGGACGGAGAAGGCTATGTTGGCCGGGCGACGGTTGTCCCGG 1464

St_rrnH TGGTGTTACTGCGAAGGGGGGACGGAGAAGGCTATGTTGGCCGGGCGACGGTTGTCCCGG 1640

Rs_RSP_4295 AGGAGGATGTGACGGATCGCAGGTGTAGT--------------------------TCGGT 1565

Go_GOX1319 TGCCAGAAGTGACGAATGAGAGATGTTGT--------------------------CTGTC 1412

Go_GOX1159 TGCCAGAAGTGACGAATGAGAGATGTTGT--------------------------CTGTC 1412

Ar_ARAD_RS00940 TGGTGGTAGTGACGGATTGCTTAACTTGT--------------------------TCACA 1481

Rl_RLEG3_RS21775 TGGTGGTAGTGACGGATTGCACAAGTTGT--------------------------TCATT 1493

Rp_RpaI_R0046 AGTGGATGGTGACGAATCCCTTATGTTGT--------------------------TCGAC 1498

Bj_BJ6T_RS07380 AGTGGATGGTGACGAATCCCGTGTGTTGT--------------------------CCGAC 1554

* **

Ec_rrlA TTTAAGCGTGTAGGCTGGTTTTCCAGGCAAATCCGGAA--AATCAAGGCTGAGGCGTGAT 1522

St_rrnH TTTAAGCGTGTAGGTGTGTGTTCCAGGTAAATCCGGTTCACTTTAACACTGAGGCGTGAC 1700

Rs_RSP_4295 CTTAT------------------------------------------------------- 1570

Go_GOX1319 CTTAA------------------------------------------------------- 1417

Go_GOX1159 CTTAA------------------------------------------------------- 1417

Ar_ARAD_RS00940 CTTAT------------------------------------------------------- 1486

Rl_RLEG3_RS21775 CTAAT------------------------------------------------------- 1498

Rp_RpaI_R0046 CTTAC------------------------------------------------------- 1503

Bj_BJ6T_RS07380 CTTAC------------------------------------------------------- 1559

* *

Ec_rrlA GACGAGGCACTACGGTGCTGAAGCAACAAATGCCCTGCTTCCAGGAAAAGCCTCTAAGCA 1582

St_rrnH GACGAGGCACTACGGTGCTGAAGCAACAAATGCCCTGCTTCCAGGAAAAGCCTCTAAGCA 1760

Rs_RSP_4295 ------------------------CGGATTGACCGGGCTGCTGAGCGGTCCCTGGAAATA 1606

Go_GOX1319 ------------------------CGGATTGAACAGGCTTTTCAATCATTCCAGGAAATA 1453

Go_GOX1159 ------------------------CGGATTGAACAGGCTTTTCAATCATTCCAGGAAATA 1453

Ar_ARAD_RS00940 ------------------------TGGATTGTGTGGGCGGGGACGCGGTTCCAGGAAATA 1522

Rl_RLEG3_RS21775 ------------------------TGGATTGGGTGGGCAGCGGAGCGGTTCCAGGAAATA 1534

Rp_RpaI_R0046 ------------------------TGGATTGGTCGGGCCTCGACGGGGTTCCAGGAAATA 1539

Bj_BJ6T_RS07380 ------------------------TGGATTGGTTGGGCTTCGAAGGGGTTCCAGGAAATA 1595

* ** ** ** *

Ec_rrlA TCAGGTAACATCAAATCGTACCCCAAACCGACACAGGTGGTCAGGTAGAGAATACCAAGG 1642

St_rrnH TCAGGTAACACGAAATCGTACCCCAAACCGACACAGGTGGTCAGGTAGAGAATACCAAGG 1820

Rs_RSP_4295 GCCCT--CCATCAGACCGTACCCCAAACCGACACAGGTGGACTGGTAGAGAATACCAAGG 1664

Go_GOX1319 GCTCTGGCGTATAGACCGTACCCGAAACCGACACAGGTGGACTGGTAGAGAATACCAAGG 1513

Go_GOX1159 GCTCTGGCGTATAGACCGTACCCGAAACCGACACAGGTGGACTGGTAGAGAATACCAAGG 1513

Ar_ARAD_RS00940 GCTCCACCGTATAGACCGTACCCGAAACCGACACAGGTGGTCAGGTAGAGTATACCAAGG 1582

Rl_RLEG3_RS21775 GCTCCACCGTATAGACCGTACCCGAAACCGACACAGGTGGTCAGGTAGAGTATACCAAGG 1594

Rp_RpaI_R0046 GCCTCC-ACATCAGACCGTACCCGAAACCGACACAGGTGGACTGGTAGAGTATACCAAGG 1598

Bj_BJ6T_RS07380 GCCTCC-ACATCAGACCGTACCCGAAACCGACACAGGTGGACTGGTAGAGTATACCAAGG 1654

* * * ******* **************** * ******* *********

Ec_rrlA CGCTTGAGAGAACTCGGGTGAAGGAACTAGGCAAAATGGTGCCGTAACTTCGGGAGAAGG 1702

St_rrnH CGCTTGAGAGAACTCGGGTGAAGGAACTAGGCAAAATGGTGCCGTAACTTCGGGAGAAGG 1880

Rs_RSP_4295 CGCTTGAGAGAACCACATCAAAGGAACTCGGCAAAATGCCTCCGTAAGTTCGCGAGAAGG 1724

Go_GOX1319 CGCTTGAGAGAACGATGCTGAAGGAACTAGGCAAATTGCTCGTGTAACTTCGGGATAAAC 1573

Go_GOX1159 CGCTTGAGAGAACGATGCTGAAGGAACTAGGCAAATTGCTCGTGTAACTTCGGGATAAAC 1573

Ar_ARAD_RS00940 CGCTTGAGAGAACTATGTTGAAGGAACTCGGCAAATTGCACGCGTAACTTCGGAAGAAGC 1642

Rl_RLEG3_RS21775 CGCTTGAGAGAACTATGTTGAAGGAACTCGGCAAATTGCACGCGTAACTTCGGAAGAAGC 1654

Rp_RpaI_R0046 CGCTTGAGAGAACTATGTTGAAGGAACTCGGCAATTTACCTCCGTAACTTCGGGATAAGG 1658

Bj_BJ6T_RS07380 CGCTTGAGAGAACTATGTTGAAGGAACTCGGCAATTTACCTCCGTAACTTCGGGATAAGG 1714

************* ******** ***** * **** **** * **

Ec_rrlA CACGCTGATATGTAGGTGAAGCGACTTGCTCGTGGAGCTGAAATCAGTCGAAGATACCAG 1762

St_rrnH CACGCTGACACGTAGGTGAAGTGATTTACTCATGGAGCTGAAGTCAGTCGAAGATACCAG 1940

Rs_RSP_4295 AGGCCCCGTCTGTAGGCAA-----------------CTATGGGCGGGGGGCACAAACCAG 1767

Go_GOX1319 GAGACCCGCTCGTGGGCAA-----------------CCATGGACGGGTGGCACAGACCAG 1616

Go_GOX1159 GAGACCCGCTCGTGGGCAA-----------------CCATGGACGGGTGGCACAGACCAG 1616

Ar_ARAD_RS00940 GTGACCCTTATCTACGCAA-----------------GTATGTGAGGGTGGCACAGACCAG 1685

Rl_RLEG3_RS21775 GTGACCCCAATCTACGCAA-----------------GTATTTTGGGGTGGCACAGACCAG 1697

Rp_RpaI_R0046 AGGCCTTCTGTTTGCGCAA-----------------GCAGGCAGGAGGGGCACAGACCAG 1701

Bj_BJ6T_RS07380 AGGCCCATTGCTCGCGCAA-----------------GCGGGCAGTGGGGGCACAGACCAG 1757

* * * * * * * *****

Ec_rrlA CTGGCTGCAACTGTTTATTAAAAACACAGCACTGTGCAAACACGAAAGTGGACGTATACG 1822

St_rrnH CTGGCTGCAACTGTTTATTAAAAACACAGCACTGTGCAAACACGAAAGTGGACGTATACG 2000

Rs_RSP_4295 GGGGTGGCGACTGTTTACTTAAAACACAGGGCTGTGCGAAGCCGCAAGGCGACGTATACA 1827

Go_GOX1319 GGGGTAGCGACTGTTTAGTAAAAACACAGGGCTCTGCGAAATCGTGAGATGACGTATAGG 1676

Go_GOX1159 GGGGTAGCGACTGTTTAGTAAAAACACAGGGCTCTGCGAAATCGTGAGATGACGTATAGG 1676

Ar_ARAD_RS00940 GGGGTAGCGACTGTTTACCAAAAACACAGGGCTCTGCGAAGTCGCAAGACGACGTATAGG 1745

Rl_RLEG3_RS21775 GGGGTAGCGACTGTTTATCAAAAACACAGGGCTCTGCGAAGTCGCAAGACGACGTATAGG 1757

Rp_RpaI_R0046 GGGGTGGCAACTGTTTAACAAAAACACAGGGCTCTGCGAAATCGCAAGATGACGTATAGG 1761

Bj_BJ6T_RS07380 GGGGTGGCAACTGTTTAACAAAAACACAGGGCTCTGCGAAATCGCAAGATGACGTATAGG 1817

** ** ******** ********* ** *** ** ** ** ********

Ec_rrlA GTGTGACGCCTGCCCGGTGCCGGAAGGTTAATTGATGGGGTTAGCCGCAAGGCGAAGCTC 1882

St_rrnH GTGTGACGCCTGCCCGGTGCCGGAAGGTTAATTGATGGGGTCAGCGC--AAGCGAAGCTC 2058

Rs_RSP_4295 GTCTGACGCCTGCCCGGTGCTGGAAGGTTAAAAGGAGGAGTGCA-----------AGCTC 1876

Go_GOX1319 GCCTGACGCCTGCCCGGTGCCGGAAGGTTAAGAGGAGGTGTGCA-----------AGCAC 1725

Go_GOX1159 GCCTGACGCCTGCCCGGTGCCGGAAGGTTAAGAGGAGGTGTGCA-----------AGCAC 1725

Ar_ARAD_RS00940 GTCTGACGCCTGCCCGGTGCTGGAAGGTTAAGAGGAGGGGTGCA-----------AGCTC 1794

Rl_RLEG3_RS21775 GTCTGACGCCTGCCCGGTGCTGGAAGGTTAAGAGGAGAGGTGCA-----------AGCTT 1806

Rp_RpaI_R0046 GTCTGACGCCTGCCCGGTGCCGGAAGGTTAAGAGGAGGAGTGCA-----------AGCTC 1810

Bj_BJ6T_RS07380 GTCTGACGCCTGCCCGGTGCCGGAAGGTTAAGAGGAGAGGTGCA-----------AGCCT 1866

* ***************** ********** * * ** ***

Ec_rrlA TTGATCGAAGCCC-CGGTAAACGGCGGCCGTAACTATAACGGTCCTAAGGTAGCGAAATT 1941

St_rrnH CTGATCGAAGCCC-CGGTAAACGGCGGCCGTAACTATAACGGTCCTAAGGTAGCGAAATT 2117

Rs_RSP_4295 CGAATTGAAGCCCCAGTAA-ACGGCGGCCGTAACTATAACGGTCCTAAGGTAGCGAAATT 1935

Go_GOX1319 TGAATTGAAGCCCCGGTAAA**C**CGGCGGCCGTAACTATAACGGTCCTAAGGTAGCGAAATT 1785

Go_GOX1159 TGAATTGAAGCCCCGGTAAA**-**CGGCGGCCGTAACTATAACGGTCCTAAGGTAGCGAAATT 1784

Ar_ARAD_RS00940 TGAATCGAAGCCCCAGTAA-ACGGCGGCCGTAACTATAACGGTCCTAAGGTAGCGAAATT 1853

Rl_RLEG3_RS21775 TGAATCGAAGCCCCAGTAA-ACGGCGGCCGTAACTATAACGGTCCTAAGGTAGCGAAATT 1865

Rp_RpaI_R0046 TGAATTGAAGCCCCGGTAA-ACGGCGGCCGTAACTATAACGGTCCTAAGGTAGCGAAATT 1869

Bj_BJ6T_RS07380 TGAATCGAAGCCCCGGTAA-ACGGCGGCCGTAACTATAACGGTCCTAAGGTAGCGAAATT 1925

** ******* * * ***************************************

Ec_rrlA CCTTGTCGGGTAAGTTCCGACCTGCACGAATGGCGTAATGATGGCCAGGCTGTCTCCACC 2001

St_rrnH CCTTGTCGGGTAAGTTCCGACCTGCACGAATGGCGTAATGATGGCCAGGCTGTCTCCACC 2177

Rs_RSP_4295 CCTTGTCGGGTAAGTTCCGACCTGCACGAATGGCGTAACGATCTCCCCGCTGTCTCTGAT 1995

Go_GOX1319 CCTTGTCGGGTAAGTTCCGACCTGCACGAATGGCGTAACGACTTCCCCACTGTCTCCAGC 1845

Go_GOX1159 CCTTGTCGGGTAAGTTCCGACCTGCACGAATGGCGTAACGACTTCCCCACTGTCTCCAGC 1844

Ar_ARAD_RS00940 CCTTGTCGGGTAAGTTCCGACCTGCACGAATGGCGTAACGACTTCCCCGCTGTCTCCAAC 1913

Rl_RLEG3_RS21775 CCTTGTCGGGTAAGTTCCGACCTGCACGAATGGCGTAACGACTTCCCCGCTGTCTCCAAC 1925

Rp_RpaI_R0046 CCTTGTCGGGTAAGTTCCGACCTGCACGAATGGCGTAATGACTTCCCCGCTGTCTCCAAC 1929

Bj_BJ6T_RS07380 CCTTGTCGGGTAAGTTCCGACCTGCACGAATGGCGTAATGACTTCCCCGCTGTCTCCAAC 1985

************************************** ** ** *******

Ec_rrlA CGAGACTCAGTGAAATTGAACTCGCTGTGAAGATGCAGTGTACCCGCGGCAAGACGGAAA 2061

St_rrnH CGAGACTCAGTGAAATTGAACTCGCTGTGAAGATGCAGTGTACCCGCGGCAAGACGGAAA 2237

Rs_RSP_4295 GTGGACTCAGCGAAATTGAACTGTGTGTCAAGATGCACACTTCCCGCGGTTAGACGGAAA 2055

Go_GOX1319 ATCGACTCAGCGAAATTGAATTCCCCGTGAAGATGCGGGGTACCCGCGGTCAGACGGAAA 1905

Go_GOX1159 ATCGACTCAGCGAAATTGAATTCCCCGTGAAGATGCGGGGTACCCGCGGTCAGACGGAAA 1904

Ar_ARAD_RS00940 ATAGACTCAGTGAAATTGAATTCCCCGTGAAGATGCGGGGTTCCTGCGGTCAGACGGAAA 1973

Rl_RLEG3_RS21775 ATAGACTCAGTGAAATTGAATTCCCCGTGAAGATGCGGGGTTCCTGCGGTCAGACGGAAA 1985

Rp_RpaI_R0046 ATAGACTCAGTGAAATTGAATTCCCCGTGAAGATGCGGGGTTCCTGCGGTCAGACGGAAA 1989

Bj_BJ6T_RS07380 ATAGACTCAGTGAAATTGAATTCCCCGTGAAGATGCGGGGTTCCTGCGGTCAGACGGAAA 2045

******* ********* * ** ******* * ** **** *********

Ec_rrlA GACCCCGTGAACCTTTACTATAGCTTGACACTGAACATTGAGCCTTGATGTGTAGGATAG 2121

St_rrnH GACCCCGTGAACCTTTACTATAGCTTGACACTGAACATTGAGCCTTGATGTGTAGGATAG 2297

Rs_RSP_4295 GACCCCATGAACCTTTACTATAGCTTCGCACTGGCATCAGGATTGTGATGTGCAGGATAG 2115

Go_GOX1319 GACCCTATGAACCTTTACTGCAGCTTTGCAGTGGCATCAGAGACATTCTGTGTAGGATAG 1965

Go_GOX1159 GACCCTATGAACCTTTACTGCAGCTTTGCAGTGGCATCAGAGACATTCTGTGTAGGATAG 1964

Ar_ARAD_RS00940 GACCCCGTGCACCTTTACTATAGCTTTACACTGGCATTCGTGTCGGCATGTGTAGGATAG 2033

Rl_RLEG3_RS21775 GACCCCGTGCACCTTTACTATAGCTTTACACTGGCATTCGTGTCGGCATGTGTAGGATAG 2045

Rp_RpaI_R0046 GACCCCGTGCACCTTTACTGTAGCTTTGCGCTGGTATTCGTGACTGTTTGTGTAGAATAG 2049

Bj_BJ6T_RS07380 GACCCCGTGCACCTTTACTGTAGCTTTGCGCTGGTATTCGTGACTGTTTGTGTAGAATAG 2105

***** ** ********* ***** * ** * **** ** ****

Ec_rrlA GTGGGAGGCTTTGAAGTGTGGACGCCAGTCTGCATGGAGCCGA-CCTTGAAATACCACCC 2180

St_rrnH GTGGGAGGCTTTGAAGTGTGGACGCCAGTCTGCATGGAGCCGA-CCTTGAAATACCACCC 2356

Rs_RSP_4295 GTGGTAGGCATCGAAGCGGGGACGCCAGTTCCCGTGGAGCCAA-CCTTGAGATACCACCC 2174

Go_GOX1319 GTCGGAGGCTTTGAAACCCAGGCGCCAGCTTGGGTGGAGCCAT-CCTTGAAATACGACCC 2024

Go_GOX1159 GTCGGAGGCTTTGAAACCCAGGCGCCAGCTTGGGTGGAGCCAT-CCTTGAAATACGACCC 2023

Ar_ARAD_RS00940 GTGGTAGGCTTTGAAGCAGGGACGCCAGTTCTTGTGGAGCCAT-CCTTGAAATACCACCC 2092

Rl_RLEG3_RS21775 GTGGTAGGCTTTGAAGCGGGGACGCCAGTTTCCGTGGAGCCAT-CCTTGAAATACCACCC 2104

Rp_RpaI_R0046 GTGGTAGGCTTTGAAGCTCGGGCGCCAGCTCGGGTGGAGCCGCAATGTGAAATACCACCC 2109

Bj_BJ6T_RS07380 GTGGTAGGCTTTGAAGCCGTGGCGCCAGCCATGGTGGAGCCGAAATGTGAAATACCACCC 2165

** * **** * *** * ****** ******* *** **** ****

Ec_rrlA TTTAATGTTTGATGTTCTAACGTTGACCCGTAATCCGGGTTGCGGACAGTGTCTGGTGGG 2240

St_rrnH TTTAATGTTTGATGTTCTAACGTGGACCCGTAATCCGGGTTGCGGACAGTGTCTGGTGGG 2416

Rs_RSP_4295 TTCGCCATCTTGATGTCTAACCGCGGCCCGTTATCCGGGTCCGGGACCCTGCGTGGTGGG 2234

Go_GOX1319 TGAATTTTTC**T**GATGTCTAACCGAGACCAGTAAGCCTGGTCCGGGACCCTGCATGGTGGG 2084

Go_GOX1159 TGAATTTTTC**C**GATGTCTAACCGAGACCAGTAAGCCTGGTCCGGGACCCTGCATGGTGGG 2083

Ar_ARAD_RS00940 TTATCGTCATGGATGTCTAACCGCGGTCCGTTATCCGGATCCGGGACAGTGTATGGTGGG 2152

Rl_RLEG3_RS21775 TTATCGTCATGGATGTCTAACCGCGGCCCGTTATCCGGGTCCGGGACAGTGTATGGTGGG 2164

Rp_RpaI_R0046 TAATGGTTATGGATATCTAACCGCGATCCCTTATCGGGTTCCGGGACAGCGCATGGTGGG 2169

Bj_BJ6T_RS07380 TAATGGTTATGGATATCTAACCGCGTCCCCTCAGCGGGGACCGGGACAGCGCATGGTGGG 2225

* ****** * * * * * * **** * *******

Ec_rrlA TAGTTTGACTGGGGCGGTCTCCTCCTAAAGAGTAACGGAGGAGCACGAAGGTTGGCTAAT 2300

St_rrnH TAGTTTGACTGGGGCGGTCTCCTCCTAAAGAGTAACGGAGGAGCACGAAGGTTGGCTAAT 2476

Rs_RSP_4295 TAGTTTGACTGGGGCGGTCGCCTCCCAAACAGTAACGGAGGCGCGCGATGGTGGGCTCAG 2294

Go_GOX1319 CAGTTTGACTGGGGCGGTCGCCTCCCAAAGTGTAA**C**GGAGGCGCGCGATGGTGGGCTCAG 2144

Go_GOX1159 CAGTTTGACTGGGGCGGTCGCCTCCCAAAGTGTAA**-**GGAGGCGCGCGATGGTGGGCTCAG 2142

Ar_ARAD_RS00940 TAGTTTGACTGGGGCGGTCGCCTCCGAAAGAGTAACGGAGGCGCGCGATGGTGGGCTCAG 2212

Rl_RLEG3_RS21775 TAGTTTGACTGGGGCGGTCGCCTCCGAAAGAGTAACGGAGGCGCGCGATGGTGGGCTCAG 2224

Rp_RpaI_R0046 CAGTTTGACTGGGGCGGTCGCCTCCCAAAGAGTAACGGAGGCGTGCGACGGTAGGCTCAG 2229

Bj_BJ6T_RS07380 CAGTTTGACTGGGGCGGTCGCCTCCCAAAGAGTAACGGAGGCGTGCGAAGGTAGGCTCAG 2285

****************** ***** *** **** ***** * *** *** **** *

Ec_rrlA CCTGGTCGGACATCAGGAGGTTAGTGCAATGGCATAAGCCAGCTTGACTGCGAGCGTGAC 2360

St_rrnH CCTGGTCGGACATCAGGAGGTTAGTGCAATGGCATAAGCCAGCTTGACTGCGAGCGTGAC 2536

Rs_RSP_4295 ACCGGTCGGAAATCGGTCGTCGAGTGCAATGGCAGAAGCCCGCCTGACTGCAAGACTGAC 2354

Go_GOX1319 GCCGGTCGGAAACCGGCTGTCGAGTGCAATGGCATAAGCCCGCCTGACTGTGAGAGTGAC 2204

Go_GOX1159 GCCGGTCGGAAACCGGCTGTCGAGTGCAATGGCATAAGCCCGCCTGACTGTGAGAGTGAC 2202

Ar_ARAD_RS00940 ACCGGTCGGAAATCGGTCGTCGAGTGCAATGGCATAAGCCCGCCTGACTGCGAGACTGAC 2272

Rl_RLEG3_RS21775 ACCGGTCGGAAATCGGTCGTCGAGTGCAATGGCATAAGCCCGCCTGACTGCGAGACTGAC 2284

Rp_RpaI_R0046 AACGGTCGGAAATCGTTCGTCGAGTACAATGGCATAAGCCTGCCTGACTGCGAGACCAAC 2289

Bj_BJ6T_RS07380 AACGGTCGGAAATCGTTCGTCGAGTATAATGGCATAAGCCTGCCTGACTGCGAGATCTAC 2345

******* * * * *** ******* ***** ** ****** ** **

Ec_rrlA GGCGCGAGCAGGTGCGAAAGCAGGTCATAGTGATCC-GGTGGTTCTGAATGGAAGGGCCA 2419

St_rrnH GGCGCGAGCAGGTGCGAAAGCAGGTCATAGTGATCC-GGTGGTTCTGAATGGAAGGGCCA 2595

Rs_RSP_4295 AAGTCGAGCAGAGACGAAAGTCGGCCATAGTGATCC-GGTGGTCCCGAGTGGAAGGGCCA 2413

Go_GOX1319 AGCTCGATCAGAGACGAAAGTCGGCCATAGTGATCC-GGTGGTCCCATGTGGACGGGCCA 2263

Go_GOX1159 AGCTCGATCAGAGACGAAAGTCGGCCATAGTGATCC-GGTGGTCCCATGTGGACGGGCCA 2261

Ar_ARAD_RS00940 AAGTCGAGCAGAGACGAAAGTCGGTCATAGTGATCC-GGTGGTCCCGCGTGGAAGGGCCA 2331

Rl_RLEG3_RS21775 AAGTCGAGCAGAGACGAAAGTCGGTCATAGTGATCC-GGTGGTCCCGCGTGGAAGGGCCA 2343

Rp_RpaI_R0046 AAGTCGAGCAGAGACGAAAGTCGGTCATAGTGATCCCGGTGGTCCCGCGTGGATGGGCCA 2349

Bj_BJ6T_RS07380 GAATCGAGCAGAGACGAAAGTCGGTCATAGTGATCC-GGTGGTCCCGCGTGGATGGGCCA 2404

*** *** ****** ** *********** ****** * **** ******

Ec_rrlA TCGCTCAACGGATAAAAGGTACTCCGGGGATAACAGGCTGATACCGCCCAAGAGTTCATA 2479

St_rrnH TCGCTCAACGGATAAAAGGTACTCCGGGGATAACAGGCTGATACCGCCCAAGAGTTCATA 2655

Rs_RSP_4295 TCGCTCAACGGATAAAAGGTACTCTGGGGATAACAGGCTGATGATGCCCAAGAGTCCATA 2473

Go_GOX1319 TCGCTCAACGGATAAAAGGTACTCTAGGGATAACAGGCTGATCTCCCCCAAGAGTCCACA 2323

Go_GOX1159 TCGCTCAACGGATAAAAGGTACTCTAGGGATAACAGGCTGATCTCCCCCAAGAGTCCACA 2321

Ar_ARAD_RS00940 TCGCTCAACGGATAAAAGGTACGCCGGGGATAACAGGCTGATGACCCCCAAGAGTCCATA 2391

Rl_RLEG3_RS21775 TCGCTCAACGGATAAAAGGTACGCCGGGGATAACAGGCTGATGACCCCCAAGAGTCCATA 2403

Rp_RpaI_R0046 TCGCTCAACGGATAAAAGGTACGCCGGGGATAACAGGCTGATGACGCCCAAGAGTCCATA 2409

Bj_BJ6T_RS07380 TCGCTCAACGGATAAAAGGTACGCCGGGGATAACAGGCTGATGACGCCCAAGAGTCCATA 2464

********************** * **************** ********* ** *

Ec_rrlA TCGACGGCGGTGTTTGGCACCTCGATGTCGGCTCATCACATCCTGGGGCTGAAGTAGGTC 2539

St_rrnH TCGACGGCGGTGTTTGGCACCTCGATGTCGGCTCATCACATCCTGGGGCTGAAGTAGGTC 2715

Rs_RSP_4295 TCGACGGCATCGTTTGGCACCTCGATGTCGGCTCATCTCATCCTGGGGCTGGAGCAGGTC 2533

Go_GOX1319 TCGACGGGGAGGTTTGGCACCTCGATGTCGGCTCATCACATCCTGGGGCTGGAGCAGGTC 2383

Go_GOX1159 TCGACGGGGAGGTTTGGCACCTCGATGTCGGCTCATCACATCCTGGGGCTGGAGCAGGTC 2381

Ar_ARAD_RS00940 TCGACGGGGTTGTTTGGCACCTCGATGTCGGCTCATCGCATCCTGGGGCTGGAGCAGGTC 2451

Rl_RLEG3_RS21775 TCGACGGGGTTGTTTGGCACCTCGATGTCGGCTCATCGCATCCTGGGGCTGGAGCAGGTC 2463

Rp_RpaI_R0046 TCGACGGCGTCGTTTGGCACCTCGATGTCGGCTCATCACATCCTGGGGCTGGAGAAGGTC 2469

Bj_BJ6T_RS07380 TCGACGGCGTCGTTTGGCACCTCGATGTCGGCTCATCACATCCTGGGGCTGGAGAAGGTC 2524

******* ************************** ************* ** *****

Ec_rrlA CCAAGGGTATGGCTGTTCGCCATTTAAAGTGGTACGCGAGCTGGGTTTAGAACGTCGTGA 2599

St_rrnH CCAAGGGTATGGCTGTTCGCCATTTAAAGTGGTACGCGAGCTGGGTTTAGAACGTCGTGA 2775

Rs_RSP_4295 CCAAGGGTATGGCTGTTCGCCATTTAAAGAGGTACGTGAGCTGGGTTTAGAACGTCGTGA 2593

Go_GOX1319 CCAAGGGTTCGGCTGTTCGCCGATTAAAGTGGTACGTGAGCTGGGTTTAGAACGTCGTGA 2443

Go_GOX1159 CCAAGGGTTCGGCTGTTCGCCGATTAAAGTGGTACGTGAGCTGGGTTTAGAACGTCGTGA 2441

Ar_ARAD_RS00940 CCAAGGGTTTGGCTGTTCGCCAATTAAAGCGGTACGTGAGCTGGGTTCAGAACGTCGTGA 2511

Rl_RLEG3_RS21775 CCAAGGGTTTGGCTGTTCGCCAATTAAAGCGGTACGTGAGCTGGGTTCAGAACGTCGTGA 2523

Rp_RpaI_R0046 CCAAGGGTTCGGCTGTTCGCCGATTAAAGTGGTACGTGAGCTGGGTTCAGAACGTCGTGA 2529

Bj_BJ6T_RS07380 CCAAGGGTTCGGCTGTTCGCCGATTAAAGTGGTACGTGAGCTGGGTTCAGAACGTCGTGA 2584

******** *********** ****** ****** ********** ************

Ec_rrlA GACAGTTCGGTCCCTATCTGCCGTGGGCGCTGGAGAACTGAGGGGGGCTGCTCCTAGTAC 2659

St_rrnH GACAGTTCGGTCCCTATCTGCCGTGGGCGCTGGAGAACTGAGGGGGGCTGCTCCTAGTAC 2835

Rs_RSP_4295 GACAGTTCGGTCCCTATCTGCCGTGGGTGTAGGAGACTTGAGAAGAGTTGCCCCTAGTAC 2653

Go_GOX1319 GACAGTTCGGTCCCTATCTGCCGTGGGTGTAAGAGACTTGAGAGGATTTGTCCCTAGTAC 2503

Go_GOX1159 GACAGTTCGGTCCCTATCTGCCGTGGGTGTAAGAGACTTGAGAGGATTTGTCCCTAGTAC 2501

Ar_ARAD_RS00940 GACAGTTCGGTCCCTATCTGCCGTGGGTGTAGGAATATTGACAGGATCTGTCCCTAGTAC 2571

Rl_RLEG3_RS21775 GACAGTTCGGTCCCTATCTGCCGTGGGTGTAGGAATATTGACAGGATCTGTCCCTAGTAC 2583

Rp_RpaI_R0046 GACAGTTCGGTCCCTATCTGCCGTGGGTGTTGGAATGTTGAGAGGATTTGCCCCTAGTAC 2589

Bj_BJ6T_RS07380 GACAGTTCGGTCCCTATCTGCCGTGGGTGTTGGAATGTTGAGAGGATTTGCCCCTAGTAC 2644

*************************** * ** *** * ** ********

Ec_rrlA GAGAGGACCGGAGTGGACGCATCACTGGTGTTCGGGTTGTCATGCCAATGGCACTGCCCG 2719

St_rrnH GAGAGGACCGGAGTGGACGCATCACTGGTGTTCGGGTTGTCATGCCAATGGCACTGCCCG 2895

Rs_RSP_4295 GAGAGGACCGGGGTGAACGATCCACTGGTGGACCAGTTGTCGTGCCAACGGCAGTGCTGG 2713

Go_GOX1319 GAGAGGACCGGGATGAACATACCTCTGGTGCACCGGTTGTCACGCCAGTGGCACAGCCGG 2563

Go_GOX1159 GAGAGGACCGGGATGAACATACCTCTGGTGCACCGGTTGTCACGCCAGTGGCACAGCCGG 2561

Ar_ARAD_RS00940 GAGAGGACCGGGATGGACATATCTCTGGTGGACCTGTTGTCCTGCCAAGGGCATAGCAGG 2631

Rl_RLEG3_RS21775 GAGAGGACCGGGATGGACATATCTCTGGTGGACCTGTTGTCCTGCCAAGGGCATAGCAGG 2643

Rp_RpaI_R0046 GAGAGGACCGGGGTGAACGTACCTCTGGTGGAGCTGTTGTCGCGCCAGCGGCAGTGCAGC 2649

Bj_BJ6T_RS07380 GAGAGGACCGGGGTGAACGTACCTCTGGTGGAGCTGTTGTCGCGCCAGCGGCAGTGCAGC 2704

*********** ** ** * ****** ****** **** **** **

Ec_rrlA GTAGCTAAATGCGGAAGAGATAAGTGCTGAAAGCATCTAAGCACGAAACTTGCCCCGAGA 2779

St_rrnH GTAGCTAAATGCGGAAGAGATAAGTGCTGAAAGCATCTAAGCACGAAACTTGCCCCGAGA 2955

Rs_RSP_4295 GTAGCTATGATCGGACAGGATAACCGCTGAAGGCATCTAAGCGGGAAGCCCCCTTCAAAA 2773

Go_GOX1319 GTAGCTAAGTATGGACGGGATAACCGCTGAAAGCATCTAAGCGGGAAACCCACCTCAAAA 2623

Go_GOX1159 GTAGCTAAGTATGGACGGGATAACCGCTGAAAGCATCTAAGCGGGAAACCCACCTCAAAA 2621

Ar_ARAD_RS00940 GTAGCTACATATGGAATGGATAACCGCTGAAGGCATCTAAGCGGGAAACCAACCTGAAAA 2691

Rl_RLEG3_RS21775 GTAGCTACATATGGACGGGATAACCGCTGAAGGCATCTAAGCGGGAAACCCACCTGAAAA 2703

Rp_RpaI_R0046 ATAGCTATGTACGGACGGGATAACCGCTGAAAGCATCTAAGCGGGAAACCCACCTCAAAA 2709

Bj_BJ6T_RS07380 ATAGCTATGTACGGACGGGATAACCGCTGAAAGCATCTAAGCGGGAAACCCACCTCAAAA 2764

****** *** ***** ****** ********** *** * * * *

Ec_rrlA TGAGTTCTCCCTGACTCCTTGAGAGTCCTGAAGGAACGTTGAAGACGACGACGTTGATAG 2839

St_rrnH TGAGTTCTCCCTGAGACTTAGAGTCTCCTGAAGGAACGTTGAAGACGACGACGTTGATAG 3015

Rs_RSP_4295 CAAGGTCTCCCT-----------------TGAGGGCCGTGGAAGACCACCACGTCGATAG 2816

Go_GOX1319 CGAGGTCTC--------------------ATAGAGCCGTGACAGACCATCACGTTGATAG 2663

Go_GOX1159 CGAGGTCTC--------------------ATAGAGCCGTGACAGACCATCACGTTGATAG 2661

Ar_ARAD_RS00940 CGAGTGTTCCCTA----------------TCAGAGCCGTGGTAGACGACCACGTTGATAG 2735

Rl_RLEG3_RS21775 CGAGTATTCCCTA----------------TCAGAGCCGTGGAAGACGACCACGTTGATAG 2747

Rp_RpaI_R0046 CGAGCATTCCCT-----------------TGAGAACCGTGGAAGACGACCACGTTGATAG 2752

Bj_BJ6T_RS07380 CGAGCATTCCCT-----------------TGAGAACCGTGGAAGACCACCACGTTGATAG 2807

** ** ** *** **** * **** *****

Ec_rrlA GCCGGGTGTGTAAGCGCAGCGATGCGTTGAGCTAACCGGTACTAATGAACCGTGAGGCTT 2899

St_rrnH GCCGGGTGTGTAAGCGCAGCGATGCGTTGAGCTAACCGGTACTAATGAACCGTGAGGCTT 3075

Rs_RSP_4295 GCCAGAGGTGTAAGCGCAGCAATGCGTTCAGCTGACTGGTACTAATTGCCCGATAGGCTT 2876

Go_GOX1319 GCCGGGTGTGAAAGTGCAGTAATGCATGCAGCTAACCGGTCCTAATCG------------ 2711

Go_GOX1159 GCCGGGTGTGAAAGTGCAGTAATGCATGCAGCTAACCGGTCCTAATCG------------ 2709

Ar_ARAD_RS00940 GCCGGGTGTGGAAGTGCGGCAACGCATGAAGCTTACCGGTACTAATAGCTCGATTGGCTT 2795

Rl_RLEG3_RS21775 GCCGGGTGTGGAAGTGCGGCAACGCATGAAGCTTACCGGTACTAATAGCTCGATCGGCTT 2807

Rp_RpaI_R0046 GCCGGATGTGGAAGTGCAGCAATGCATGTAGCTTACCGGTACTAATCGTTCGATTGGCTT 2812

Bj_BJ6T_RS07380 GCCGGGTGTGGAAGTGCAGTAATGCATGCAGCTTACCGGTACTAATCGTTCGATTGGCTT 2867

*** * *** *** ** * * ** * **** ** *** *****

Ec_rrlA AACCTT------------ 2905

St_rrnH AACCTTACAACGCCGAAG 3093

Rs_RSP_4295 GATCT------------- 2881

Go_GOX1319 ------------------ 2711

Go_GOX1159 ------------------ 2709

Ar_ARAD_RS00940 GATTGTTCTCATT----- 2808

Rl_RLEG3_RS21775 GATCGTTCTCATT----- 2820

Rp_RpaI_R0046 GATTGCTCTCATTAT--- 2827

Bj_BJ6T_RS07380 GATTGCTCTCATTTT--- 2882

CLUSTAL O(1.2.4) multiple sequence alignment

**Figure S2** Alignment of 23S rRNA gene sequences from *E. coli*MG1655 (Ec_rrlA), *Salmonella typhimurium* LT2 (St_rrnH), *Rhodobacter sphaeroides* 2.4.1 (Rs_RSP_4295), *G. oxydans* 621H (Go_GOX1319 and Go_GOX1159), *Agrobacterium radiobacter* K84 (Ar_ARAD_RS00940), *Rhizobium leguminosarum bv. trifolii* WSM1689 (Rl_RLEG3_RS21775), *Rhodopseudomonas palustris* TIE-1 (Rp_RpaI_R0046), *Bradyrhizobium japonicum* USDA 6 (Bj_BJ6T_RS07380). Fragmentation positions in rRNAs known or assumed for other bacteria are underlined according to the literature. Known RNase III cleavage sites are framed. Regions in *G. oxydans* which exhibited read coverage <5% of the average gene coverages (Figure 6) are highlighted in light grey (found in all four rRNA genes) and dark grey (found only in GOX1159). Differences between the rRNA sequences GOX1319 and GOX1159 are underlined and bolded. Boxes at these positions indicate identical base in GOX0221 and GOX1467.
